# Oncogenic KRAS mutation confers chemoresistance by upregulating SIRT1 in non-small cell lung cancer

**DOI:** 10.1038/s12276-023-01091-0

**Published:** 2023-10-02

**Authors:** Dong Hoon Shin, Jeong Yeon Jo, Minyoung Choi, Kyung-Hee Kim, Young-Ki Bae, Sang Soo Kim

**Affiliations:** 1 Research Institute, National Cancer Center, Goyang-si, Gyeonggi-do Republic of Korea; 2 Cancer Biomedical Science, Graduate School of Cancer Science and Policy, National Cancer Center, Goyang-si, Gyeonggi-do Republic of Korea

**Keywords:** Cancer therapeutic resistance, Oncogenes

## Abstract

Kirsten rat sarcoma viral oncogene homologue (KRAS) is a frequent oncogenic driver of solid tumors, including non-small cell lung cancer (NSCLC). The treatment and outcomes of KRAS-mutant cancers have not been dramatically revolutionized by direct KRAS-targeted therapies because of the lack of deep binding pockets for specific small molecule inhibitors. Here, we demonstrated that the mRNA and protein levels of the class III histone deacetylase SIRT1 were upregulated by the KRAS^Mut^-Raf-MEK-c-Myc axis in KRAS^Mut^ lung cancer cells and in lung tumors of a mouse model with spontaneous Kras^G12D^ expression. KRAS^Mut^-induced SIRT1 bound to KRAS^Mut^ and stably deacetylated KRAS^Mut^ at lysine 104, which increased KRAS^Mut^ activity. SIRT1 knockdown (K/D) or the SIRT1^H363Y^ mutation increased KRAS^Mut^ acetylation, which decreased KRAS^Mut^ activity and sensitized tumors to the anticancer effects of cisplatin and erlotinib. Furthermore, in Kras^G12D/+^;Sirt1^co/co^ mice, treatment with cisplatin and erlotinib robustly reduced the tumor burden and increased survival rates compared with those in spontaneous LSL-Kras^G12D/+^;Sirt1^+/+^ mice and mice in each single-drug treatment group. Then, we identified p300 as a KRAS^Mut^ acetyltransferase that reinforced KRAS^Mut^ lysine 104 acetylation and robustly decreased KRAS^Mut^ activity. KRAS^Mut^ lysine 104 acetylation by p300 and deacetylation by SIRT1 were confirmed by LC‒MS/MS. Consistent with this finding, the SIRT1 inhibitor EX527 suppressed KRAS^Mut^ activity, which synergistically abolished cell proliferation and colony formation, as well as the tumor burden in KRAS^Mut^ mice, when combined with cisplatin or erlotinib. Our data reveal a novel pathway critical for the regulation of KRAS^Mut^ lung cancer progression and provide important evidence for the potential application of SIRT1 inhibitors and p300 activators for the combination treatment of KRAS^Mut^ lung cancer patients.

## Lung cancer: Protein interaction as a target of chemotherapy

The interaction between two key proteins in non-small cell lung cancers (NSCLCs) could be targeted to improve the success of chemotherapy. Mutations in the KRAS protein, a regulator of cell growth, are a common driver of NSCLCs, and are associated with poor prognosis. Treating NSCLCs harboring KRAS mutations has proven challenging, and further insights into the mechanisms driving KRAS activity are needed. Using cell cultures and mouse models, Dong Hoon Shin at the National Cancer Center in Goyang-si, South Korea, and co-workers demonstrated that KRAS mutations confer resistance to chemotherapy in NSCLC cells. KRAS mutations increase levels of a protein called SIRT1, and this interaction boosts cancer cell proliferation and tumour growth. Knocking out SIRT1 improved the success of chemotherapy in mouse models. Combined therapies involving SIRT1 inhibitors may therefore improve treatments for NSCLCs.

## Introduction

Lung cancer is the most common cause of cancer-related mortality worldwide^1^. Non-small cell lung cancer (NSCLC) accounts for over 85% of all cases of lung cancer^2^. NSCLCs are subclassified as adenocarcinoma (approximately 40% of all lung cancers), squamous cell carcinoma (approximately 35% of all lung cancers), large cell carcinoma (approximately 10% of lung cancers), and other rarer subtypes^3^. Most patients with NSCLC have advanced-stage disease with treatment limited to surgery, chemotherapy, and/or radiation therapy. These are associated with a high risk of tumor recurrence and poor overall 5-year relative survival rates^2,4^.

One of the most frequent driver mutations of NSCLCs is *KRAS*^*Mut*^ (present in approximately 8–24% of cases)^5^. Generally, mutations in the *KRAS* gene occur most frequently at codons 12, 13, and 61 (G12X, G13X, and Q61X, respectively), and they activate KRAS signaling by either impairing the intrinsic GTPase activity of KRAS or structurally altering KRAS such that it becomes insensitive to GTPase-activating proteins. Therefore, the activity of signaling cascades stimulated by *KRAS*^*Mut*^ enhances proliferation, apoptosis, and differentiation^6,7^. The definitive role of *KRAS*^*Mut*^ in the molecular pathogenesis of lung adenocarcinoma is demonstrated by the spontaneous development of lung adenocarcinoma in mouse models genetically engineered to conditionally express the oncogenic *KRAS*^*G12D*^ allele in the airway epithelium^8^.

Recently, attempts to directly target *KRAS*^*Mut*^ have been made with approaches similar to those targeting ATP-binding proteins. The development of competitive inhibitors instead of agents binding GTP into KRAS has been challenging because of the picomolar binding affinity between KRAS and GTP and the abundances of both GDP and GTP in cells^9^. In addition, there is no direct *KRAS*^*Mut*^ inhibitor with activity against all types of *KRAS*^*Mut*^ even at the bench level. Therefore, Shokat and colleagues engineered the drugs sotorasib (AMG510) and adagrasib (MRTX849) to be highly specific, irreversible small molecule allosteric inhibitors of *KRAS*^*G12C*^ that maintain it in the inactive state^10^. Unfortunately, a response to KRAS^G12C^ inhibitors was lacking in the majority of KRAS^G12C^ NSCLC patients. These results indicate that inhibitors directly targeting KRAS^G12C^ can be subject to intrinsic resistance, whose mechanisms include signaling rebound and adaptive changes in the signaling network. KRAS^G12C^ inhibitors suppress MAPK signaling for a short duration, and signaling rebound has been observed in both cell models and mouse xenograft models within 24 to 72 h of treatment^11,12^.

Direct targeting of *KRAS*^*Mut*^ lung cancers has proven difficult. Over the past several years, approaches for indirect targeting of *KRAS*^*Mut*^ have been challenged due to the posttranslational modification, localization, and upstream activation of KRAS and the blockade of downstream effector pathways^13^. Among these limiting factors, we focused on KRAS posttranslational modification. KRAS protein function is tightly regulated by posttranslational modification. For example, the KRAS splice isoforms KRAS4A and KRAS4B are farnesylated on a C-terminal cysteine, and only KRAS4A is subsequently palmitoylated^14^. KRAS, upon regulation by posttranslational modifications, impacts its downstream effectors, which are associated with the plasma membrane. In addition, other posttranslational modifications directly regulate KRAS activity. For example, lysine 147 in KRAS can be monoubiquitinated, which enhances guanosine triphosphate (GTP) binding and thus enhances the binding of KRAS to Raf and phosphatidylinositol 3-kinase^15^. Importantly, KRAS acetylation on lysine 104 has been reported to reduce guanine nucleotide exchange factor (GEF)-mediated nucleotide exchange, making it difficult to reload guanine triphosphate (GTP)^16^. Therefore, if the acetylation of *KRAS*^*Mut*^ can be maintained by reducing the deacetylation of the KRAS mutant protein, this would be an efficient regulatory method to attenuate the transforming activity of KRAS. Interestingly, we found that the mRNA and protein levels of SIRT1 were increased in KRAS^Mut^ cell lines and the *Kras*^*LA2_G12D*^ mouse model. Therefore, we thought that increased SIRT1 expression has a promoting role in *KRAS*^*Mut*^ cancer by reducing the acetylation of *KRAS*^*Mut*^, which results in the activation of KRAS^Mut^.

Sirtuin family proteins are class III HDACs and homologs of the yeast Sir2 protein^17^. The sirtuin family member SIRT1 can deacetylate both histones and non-histone proteins such as p53^18^. Its deacetylase activity can regulate gene transcription and affect a variety of cellular processes, such as aging^19^, apoptosis^20^, inflammation^21^, stress resistance^21,22^, and metabolism^23^. *SIRT1* plays a role in tumor progression by acting as either a tumor suppressor or oncogene^24^. Interestingly, SIRT1 does not exhibit a fixed expression level. For example, SIRT1 expression has been found to be decreased in human breast, liver, and colon tumors^25,26^. Conversely, increased SIRT1 expression has been correlated with favorable outcomes in human head and neck squamous cell carcinomas and ovarian tumors^27,28^. However, the reverse is also true. Reduced SIRT1 expression was found to accelerate tumor development in a p53-heterozygous background^25^, and its overexpression was shown to block the growth of different types of tumors in cell culture experiments^29,30^ and mouse models^31^. *SIRT1* can also act as an oncogene. For example, SIRT1 levels have been found to be increased in several human tumors, such as breast^32^, prostate^33^, liver^34^, leukemia^35^, and NSCLC^36,37^. Overexpression of Sirt1 promotes thyroid tumor formation in mice^38^ and SIRT1 downregulation decreases intestinal tumor formation^39^. In addition, another report showed that SIRT1 protein levels were decreased in mouse embryonic fibroblasts (MEFs) overexpressing KRAS and in two human lung adenocarcinoma cell lines. In addition, the size of carcinoma tumors but not adenoma tumors in Kras^Tg^;Sirt1^Tg^ mice was reduced compared with that in Kras^Tg^;Sirt1^WT^ mice^40^.

Taken together, these observations indicate that *KRAS*^*Mut*^ is relatively strongly associated with poor prognosis compared to other mutations, such as *EGFR* mutations, and participates in a mechanism of resistance to platinum-based or EGFR tyrosine kinase inhibitor (TKI)-based chemotherapeutic strategies^41,42^. Despite these observations, the development and clinical implementation of specific KRAS inhibitors have proven technically challenging, and there is a pressing need to identify druggable cooperating partners for oncogenic *KRAS*^43^. The functional interaction between SIRT1 and KRAS remains poorly understood. Although it has been reported that SIRT1 binds to the promoters of RAS genes^44,45^ and directly binds and deacetylates KRAS at lysine 104, promoting KRAS activation^16^, the mechanisms of these interactions are not yet fully understood. In this study, we performed biochemical analyses to investigate the functional interactions between SIRT1 and KRAS^Mut^. We also confirmed that SIRT1 deacetylates KRAS^Mut^ at lysine 104 in KRAS^Mut^ cells and that SIRT1 expression is reciprocally increased by KRAS^Mut^ activation. We identified a strong positive feedback loop between KRAS^Mut^ and SIRT1. Therefore, inactivating the positive feedback loop between KRAS^Mut^ and SIRT1 by reducing the SIRT1 level could be an attractive therapeutic strategy for *KRAS*^*Mut*^ cancer.

## Materials and methods

### Cell lines and culture conditions

The H358, H460, NCIH23, SKLU-1, SW960, A427, H727 (KRAS^Mut^), H1650 (KRAS and EGFR double mutant), H1975, HCC827, HCC2279 (EGFR^Mut^), HCC1666, H322M, H522, Calu-3, H1299 (KRAS and EGFR wild type), BEAS-2B (nontumorigenic lung epithelial cells), CCD-18Lu (nontumorigenic lung fibroblasts), and human embryonic kidney (HEK) 293 T cell lines were obtained from the American Type Culture Collection (Manassas, VA, USA). The GFP-positive KRAS^G12C^ mutant stable cell lines H1299 KRAS^G12C_K104^, H1299 KRAS^G12C_K104R^, and H1299^H363Y^ were generated by stably cotransfecting H1299 KRAS^WT^ cells. The cells were cultured in either HyClone RPMI-1640 medium (Thermo Fisher Scientific, Waltham, MA, USA) or Dulbecco’s modified Eagle’s medium (Thermo Fisher Scientific, Waltham, MA, USA), each containing 10% FBS and 1% penicillin and streptomycin. Cells were maintained in a 37 °C humidified incubator with 5% CO_2_ in air. All cell lines were authenticated by short tandem repeat (STR) analysis and were free of mycoplasma contamination.

### Plasmids, antibodies, and assay kits

Plasmids such as *KRAS*^*WT*^ (#75282), *KRAS*^*G12C*^ (#58901), *SIRT1* (#13735), *Flag-SIRT1*^*H363Y*^ (#1792), and *Flag-p300* (#23252) were purchased from Addgene (Watertown, MA, USA). Small interfering RNAs (siRNAs), including siKRAS (sc-35731 and sc-43874), siSIRT1 (sc-40986), siSIRT2 (sc-40988), siHDAC6 (sc-35544), sip300 (sc-29431), sic-Myc (sc-29226), siADAM17 (sc-36604), and shSIRT1 (sc-40986), and antibodies, including antibodies specific for SIRT1 (sc-15454), SIRT2 (sc-20966), KRAS (F234, sc-30), Tubulin (sc-32293), PARP (sc-8007), cleaved PARP (sc-56196), and p300 (sc-32244), were purchased from Santa Cruz Biotechnology Inc. (Dallas, TX, USA). Antibodies against GFP (#2555), ERK1/2 (#4695), phospho-ERK (T202/Y204) (#4370), phospho-Akt (S473) (#4060), Akt (#4685), cleaved caspase-3 (Asp175) (#9661), and β-actin (#3700) were obtained from Cell Signaling Technology Inc. (Danvers, MA, USA). Anti-acetylated lysine (ab190479), anti-TATA binding protein (TBP) (ab818), anti-phospho-c-Myc (ab51156), anti-c-Myc (ab32072), anti-ki67 (ab92742), anti-phospho ADAM17 (T735) (ab182630), anti-ADAM17 (ab28233) antibodies and the terminal deoxynucleotidyl transferase dUTP nick end labeling (TUNEL) assay kit-HRP-DAB (ab206386) were obtained from Abcam (Cambridge, MA, USA). The KRAS activation assay kit (STA-400-K) was obtained from Cell Biolabs Inc. (San Diego, CA, USA). Erlotinib (S7786), cisplatin (S1166), LY294002 (S1105), and EX527 (S1541) were purchased from Selleck Chemicals (Houston, TX, USA). Additionally, a human HB-EGF ELISA kit (DHBEG0) and a human Amphiregulin ELISA kit (DAR00) were purchased from R&D Systems (Abingdon, OX, UK).

### Transfection and establishment of stable cell lines

For transient overexpression or knockdown, H358, H1299, A427, H727, H23 and HEK293T cells at 40% confluence were transfected with plasmids (2 μg per 60-mm dish) or siRNAs (80 nM) using Lipofectamine^®^ 2000 (Thermo Fisher Scientific, Waltham, MA, USA). Transfected cells were allowed to stabilize for 48 h prior to use in experiments.

### Cell viability and colony formation assays

For the cell viability assay, 2 × 10^3^ cells were plated in 96 well plates and incubated with medium containing the drugs for six days. Cell viability was measured using CellTiter 96^®^ AQueous One Solution Reagent (Promega, Madison, WI, USA) containing a tetrazolium compound [3-(4,5-dimethylthiazol-2-yl)-5-(3-carboxymethoxyphenyl)-2-(4-sulfophenyl)-2H-tetrazolium, inner salt; MTS] at 490 nm for 90 min every third day (VersaMax, San Jose, CA, USA). Six replicate wells were used for each analysis, and at least three independent experiments were performed. To analyze anchorage-independent growth, cells (2 × 10^3^ cells/well) were suspended in 0.4% top agar and cultured on 0.8% agar for 40 days. The cells were stained with crystal violet, and cell masses (0.2-mm diameter) were counted as colonies.

### Quantitative real-time PCR

Total cellular RNA was extracted with TRIzol (Invitrogen Life Technologies, USA) using chloroform, precipitated with isopropyl alcohol, washed with 70% ethanol, and eluted in RNase-free water. The concentration of the isolated RNA was measured using a Nanodrop 2000 spectrophotometer (Thermo Scientific, USA) at 260 nm. The cDNAs used as templates in RT‒qPCR were prepared using 1000 ng of total RNA. mRNA expression was evaluated by RT‒qPCR using Sensi FAST SYBR (Bioline) and normalized to RPL32 expression in each sample. For PCR, DNA polymerase activation was performed for 5 min at 94 °C, and amplification was then conducted in a Light Cycler 96 instrument (Roche Diagnostics, Switzerland). The primer sequences used in these experiments are listed in Table 1.

**Table 1:** Primer sequences used for quantitative real-time PCR.

Gene	Forward Primer	Reverse Primer
*SIRT1*	TAGCCTTGTCAGATAAGGAAGGA	TGTTCTGGGTATAGTTGCGAAGT
*Sirt1*	GGAGCAGATTAGTAAGCGGCTTG	GTTACTGCCACAGGAACTAGAGG
*SIRT2*	TGCGGAACTTATTCTCCCAGA	GAGAGCGAAAGTCGGGGAT

All reactions were carried out in triplicate. The comparative threshold cycle (ΔCT) method was used to compare 2-ΔΔCT values.

ΔCT = CT (target gene) – CT (reference gene)

ΔΔCT = ΔCT (target sample) - ΔCT (reference sample)

### KRAS activation assay

Active GTP-bound KRAS was quantified using a KRAS activation assay kit (Catalog No. STA-400-K, Cell Bio Labs, San Diego, CA, USA) according to the manufacturer’s instructions. Briefly, 1 ml of total cell lysate (2.0 mg) was incubated with 40 μl Raf1-RBD agarose beads for 2 h at 4 °C to pull down the activated GTP-bound KRAS. The bead pellet was washed three times with 0.5 ml of 1× assay buffer, with centrifugation and aspiration each time. After the last wash, the bead pellet was resuspended in 40 μl of 2× reducing sodium dodecyl sulfate‒polyacrylamide gel electrophoresis (SDS‒PAGE) sample buffer. The samples were electrophoresed, proteins were transferred to a PVDF membrane, and the membrane was incubated with an anti-KRAS antibody (Abnova, Walnut, CA, USA, Catalog No. H00003845-M01) to measure the KRAS protein level.

### ChIP-Seq analysis

Chromatin immunoprecipitation (ChIP) assays were conducted using the EpiTect ChIP OneDay kit (QIAGEN, Catalog No. 334471) according to the manufacturer’s protocol. Cells were fixed with 1% formaldehyde. Chromatin was sonicated and precleared using protein A beads. Precleared chromatin solutions were incubated with 2 μg of specific anti-KRAS (Santa Cruz, sc-30), anti-SIRT1 (Santa Cruz, sc-15454), anti-SIRT2 (Santa Cruz, sc-20966) antibodies or with immunoglobulin G (IgG) on a rotating platform at 4 °C overnight. Antibody-enriched protein‒DNA complexes were dissociated by reversal of crosslinking and purified according to the manufacturer’s instructions. qPCR analysis was conducted on the immunoprecipitated DNA fractions. The KRAS antibody used in this study was purchased from Novus (Novus, Catalog No. H00003845-P01), and normal mouse IgG was purchased from Santa Cruz Biotechnology (Santa Cruz, Catalog No. sc-2025). The primer sequences used in these experiments are listed in Table 2.

**Table 2:** Primer sequences used for chromatin immunoprecipitation (ChIP)-PCR.

Gene	Forward Primer	Reverse Primer
*KRAS*	GCGCTGACCTAGGGAATGTT	TCACTTCACAGCACGTACTCC
*SIRT1*	TGACCGATGGACTCCTCACT	GCGTGTGACGTTCTGTCATC
*SIRT2*	GCACTGGGCCCTCTTAACAT	CTGGGATTGAGTTGGGGACC

### In vitro acetylation and deacetylation assay

Freshly prepared recombinant hKRAS^G12C^ protein (SignalChem, #R06–32DH) and the hp300 catalytic domain (C.D.) (Enzo, BML-SE451) were incubated in the reaction mixture [50 mmol/L Tris-HCl (pH 8.0), 0.1 mmol/L EDTA, 1 mM DTT, 10% glycerol, and 10 mmol/L acetyl-CoA (Merck, #10101893001)] at 30 °C. When needed, after incubation, the hp300 C.D. was cleaved by overnight incubation with thrombin at 4 °C. After the in vitro acetylation assay, the acetylated KRAS^G12C^ and recombinant hSIRT1 proteins (Abcam, ab54334) were incubated in deacetylation buffer [50 mM NaCl, 50 mM Tris-HCl (pH 9.0), 4 mM MgCl_2_, 5% glycerol, 0.5 mM dithiothreitol, 0.02% NP-40, and 0.1 mM PMSF] supplemented with 50 μM NAD^+^ at 30 °C for 2 h. Proteins were separated by SDS‒PAGE, and the level of KRAS^G12C^ acetylation was evaluated with an anti–acetylated lysine (Ac-K) antibody.

### Mass spectrometry

Acetylated residues were identified by mass spectrometry performed at the Proteomics and Mass Spectrometry (PAMS) facility at the National Cancer Center of South Korea. The protein products of acetylation reactions, as described above, were separated by SDS‒PAGE. The 23.1 kDa KRAS band was excised and subjected to in-gel tryptic digestion. The digested peptides were analyzed using a Q ExactiveTM Plus hybrid quadrupole-Orbitrap mass spectrometer (Thermo Fisher Scientific, Waltham, MA, USA) coupled to an UltiMate 3000 RSLCnano system (Thermo Fisher Scientific, Waltham, MA, USA). The eluted peptides were sprayed onto a nano-ESI source at an electrospray voltage of 2.0 kV. The Q Exactive Plus mass analyzer was operated using the top 10 data-dependent method. Full MS scans were acquired over the range of m/z 350–2000 with a mass resolution of 70,000 (at m/z 200). The AGC target value was 3.00E + 06. The ten most intense peaks with a charge state ≥2 were fragmented in the higher-energy collisional dissociation (HCD) collision cell with a normalized collision energy of 27, and tandem mass spectra were acquired in the Orbitrap mass analyzer with a mass resolution of 17,500 at m/z 200.

Database search—Database searching for all raw data files was performed using IP2 (Integrated Proteomics Pipeline) software (Bruker Corporation). ProLuCID was used for database searching against the SwissProt human database. Database searching against the corresponding reverse database was performed to evaluate the false discovery rate (FDR) of peptide identification. The database search parameters included a precursor ion mass tolerance of 10 ppm, fragment ion mass tolerance of 100 ppm, fixed modification of carbamidomethyl cysteine and variable modifications of methionine oxidation and lysine acetylation. We obtained an FDR of less than 1% at the peptide level and performed filtering to select the peptides with high confidence.

### Combination index calculation

The percentage of surviving cells in each drug-treated group relative to that in the DMSO-treated cell group as the control group, which was defined as 100% viability, was estimated. Data obtained from the cell viability assays were used to perform these analyses. The observed dose combination of the two agents that achieved a particular Fa was then plotted on an isobologram. The combination index (CI) is a mathematical and quantitative representation of a two-drug pharmacological interaction. Using data from the cell viability assay and computerized software, CI values were generated over a range of Fa values for treatment with EX527 and a combination of erlotinib and cisplatin. Analysis of the combinational effect was performed using the CompuSyn program (Chou and Talalay). CompuSyn was used to compute the CI for each drug combination studied using cell viability assays. The CI indicates whether the effect of a drug combination is additive (CI = 1), synergistic (CI < 1), or antagonistic (CI > 1). This program also provides algorithms for in silico simulation of synergism and/or antagonism at any combination of concentrations, as presented in the CI plot^46^.

### Apo-BrdU TUNEL assay

Cells treated with different concentrations of drugs were harvested and processed for flow cytometry (FACS Fortessa, Becton Dickinson) using the APO-BrdUTM TUNEL Assay Kit with Alexa Fluor® 488 Anti-BrdU (Molecular Probes, Invitrogen Detection Technologies, USA, Catalog No. A23210) according to the manufacturer’s instructions. Terminal deoxynucleotide transferase dUTP nick-end labeling (TUNEL) is a method for detecting DNA fragments during the cell cycle by labeling the 3′-hydroxyl termini of DNA double-strand breaks. Floating and attached cells were fixed with 4% paraformaldehyde and subsequently incubated with a DNA-labeling solution containing BrdU. After washing, the samples were incubated with an Alexa Fluor-conjugated anti-BrdU antibody, pelleted, and washed with wash buffer. The cells were incubated with RNase/propidium iodide (PI) solution (provided in the kit) at 37 °C for 15 min. The cells were analyzed by flow cytometry, and the values were expressed as the percentage of apoptotic cells (among total cells). Each experiment was performed in triplicate.

### PI-Annexin V apoptosis assay

Apoptosis was also evaluated using annexin V-PI staining followed by flow cytometry. After treatment with different combinations of drugs, cells were collected and centrifuged at 300 × g for 5 min and then washed twice with cold PBS. The cells were then resuspended in binding buffer and incubated with PI and annexin V-FITC (BD Biosciences, Catalog No. RUO-556547) for 15 min at 21 °C. A minimum of 10,000 events were collected and analyzed by flow cytometry using a BD LSR Fortessa cell analyzer (Becton Dickinson, USA).

### Immunoblotting and immunoprecipitation

Proteins obtained from the cell extracts were separated by sodium dodecyl sulfate‒polyacrylamide gel electrophoresis (SDS‒PAGE) and then transferred to Immobilon-P membranes (Millipore, Bedford, MA, UK). The membranes were blocked with 5% nonfat milk, incubated overnight at 4 °C with primary antibodies diluted 1:100–1:1000, and then incubated for 1 h at room temperature (24–26 °C) with the corresponding horseradish peroxidase-conjugated secondary antibodies. Antigen-antibody complexes were visualized using SuperSignal West Femto luminol enhancer solution (Thermo Fisher Scientific, Waltham, MA, USA). For immunoprecipitation, cell lysates were incubated with 5 μl of an anti-KRAS-GTP-bound antibody, anti-EGF antiserum, or preimmune serum at 4 °C for 2 h. Immune complexes were further incubated with protein A/G-Sepharose beads (GE Healthcare, Chicago, IL, USA) at 4 °C for 4 h. The immune complexes were eluted by boiling for 10 min in sample buffer containing 2% SDS and 10 mM dithiothreitol, subjected to SDS‒PAGE, and then analyzed by immunoblotting using anti-rabbit and anti-mouse antibodies.

### Immunohistochemical (IHC) staining

Paraffin-embedded Section (4 μm thick) were deparaffinized, and heat-induced epitope retrieval was performed using targeted retrieval solution 9 (Dako, Santa Clara, CA, USA). The slides were treated with 3% hydrogen peroxide for 20 min to block endogenous peroxidase activity prior to washing with deionized water for 2–3 min. The slides were then incubated first with 0.5% BSA blocking solution for 1 h at room temperature and then with primary antibodies against pERBB2 (Y1221/1222), pERBB3 (Y1289), pERK (T202/Y204), pAKT (S473), and cleaved caspase-3 and antibodies provided in the TUNEL assay kit overnight at 4 °C. Immunoreactions were detected using a VECTASTAIN^®^ ABC HRP Kit (Burlingame, CA, USA). Hematoxylin was used for counterstaining.

### Human HB-EGF and Amphiregulin immunoassay

Microplate strips were removed from the plate frame, and 100 µl of assay diluent buffer was added to each well. Standard, control, and test samples were added at 100 µl per well, and the tubes were covered with the adhesive strip and incubated for 2 h at 4 °C. The buffer and cell culture supernatant in each tube were aspirated and washed with 400 µl of wash buffer, and these steps were repeated three times. Human HB-EGF and Amphiregulin conjugate (200 µl) were added to each well, and the tubes were incubated for 2 h at 4 °C and washed with wash buffer three times. Substrate solution (200 µl) was added to each tube, and the tubes were incubated for 30 min at room temperature protected from light. The plate was gently tapped, and the color change from blue to yellow was observed in the wells. Then, 50 µl of stop solution was added to each well. The optical density was measured over a duration of 30 min at a wavelength of 450 nm (VERSAmax, San Jose, CA, USA).

### Orthotopic lung cancer mouse model

All animal procedures were performed in accordance with a protocol approved by the Institutional Animal Care and Use Committee (IACUC) of the National Cancer Centre Research Institute (NCCRI). NCCRI is an Association for Assessment and Accreditation of Laboratory Animal Care International (AAALAC International)-accredited facility that abides by the guidelines of the Institute of Laboratory Animal Resources (ILAR) Guide and Usage Committee. Nude mice (BALB/cAnN.Crj-nu/nu) obtained from Charles River Laboratories Japan (CRLJ, Shin-Yokohama, Japan) were anesthetized with isoflurane via inhalation in an enclosed box chamber. Mice were placed in a supine position, and the jaw and tongue were drawn away from the esophageal region using forceps while a 22-gauge Hamilton TLC syringe (Model # 1705, Hamilton, Reno, NV) was inserted into the trachea. The upper chest of the mouse was illuminated, and the mouse was injected with 1 × 10^6^ cancer cells suspended in 100 μl of PBS. After the injection, the mouse was allowed to recover before being returned to its cage for a predetermined period after exposure.

### LSL-Kras^G12D/+^;Sirt1^+/+^ and LSL-Kras^G12D/+^;Sirt^co/co^ mouse models

LSL-Kras^G12D/+^ (B6.129S4-*Kras*^*tm4Tyj*^/J, stock #008179) mice on a C57BL/6 J background and Sirt1^co/co^ (B6.129-*Sirt1*^*tm1guj*^/J, stock #008041) mice were purchased from The Jackson Laboratory (Sacramento, CA, USA). LSL-Kras^G12D/+^;SIRT1^co/co^ mice were generated by crossing LSL-Kras^G12D/+^;SIRT1^+/+^ mice with LSL-Kras^G12D/+^;SIRT1^co/co^ mice. First-generation LSL-Kras^G12D/+^;SIRT1^co/+^ mice were backcrossed with Kras^+/+^;SIRT1^co/co^ mice to generate LSL-Kras^G12D/+^;SIRT1^co/co^ mice. Initially, LSL-Kras^G12D/+^;SIRT1^+/+^ and LSL-Kras^G12D/+^;SIRT1^co/co^ mice were anesthetized with isoflurane via inhalation in an enclosed box chamber and infected with 5 × 10^7^ infectious Ad-Cre particles (HanBio, South Korea) per mouse via intranasal injection.

### Kras mutation transgenic mouse model

All animal procedures were conducted in accordance with the protocol approved by the National Cancer Center of Korea. Kras^LA2(G12D)^ (129 S/Sv-*Kras*^*tm3Tyj*^/J, stock #008185) mice were purchased from The Jackson Laboratory. Homozygosity for the Kras^LA2(G12D)^ allele is lethal in mice. Heterozygous mice develop tumors in the lungs with 100% incidence; these tumors are first detectable as pleural nodules at eight weeks after birth.

### FDG-positron emission tomography/computed tomography (PET/CT) scanning

The mice in this study were assigned to the LSL-Kras^G12D/+^;Sirt1^+/+^ and LSL-Kras^G12D/+^;Sirt1^co/co^ age-matched groups. PET/CT scanning was performed after the mice were fasted for 12 h with free access to water. Mice were anesthetized using vaporized isoflurane (4% for induction; 2.5% for maintenance). Sterile normal saline (0.1 ml) was subcutaneously injected to ensure adequate hydration. PET/CT scanners (Biograph LOS, Siemens Healthcare, Erlangen, Germany; Discovery LS, GE Healthcare, Milwaukee, WI, USA). Noncontrast CT images were acquired in a region ranging from the skull base to the upper thigh, and subsequent PET images were acquired 60 min after lateral tail vein injection of 18F-FDG (8.65 ± 2.7 MBq). The standardized uptake value (SUV) was calculated as (decay-corrected activity [kBq] per mL of tissue volume)/body mass [g]). The SUVs of lesions were calculated by manually placing a volume of interest (VOI) around the lesion. Dynamic spiral CT imaging was performed using a multidetector CT scanner (Lightspeed pro-16, GE Healthcare) with contrast enhancement.

### Prediction of deacetylation and the shortest path between histone deacetylases and KRAS

Deacetylation of the GTPase KRAS was predicted using the acetylation set enrichment-based (ASEB) method, which identifies novel lysine acetyltransferase (KAT)- or HDAC-specific acetylation and deacetylation sites based on the different characteristics of sets of lysine sites^47^. Sequence information of KRAS (UniProt accession number, P01116) was used to predict deacetylation sites targeted by HDAC1/HDAC2/HDAC3 and SIRT1. The P values for the query peptides were between 0.0001 and 1 (http://bioinfo.bjmu.edu.cn/huac/predict_p/).

The shortest path between and protein interaction network for KRAS and SIRT1 were also estimated using the PINA (Protein Interaction Network Analysis, http://cbg.garvan.unsw.edu.au/pina/) platform and STRING (Search Tool for the Retrieval of Interacting Genes/Proteins, http://string-db.org/) database via the ASEB method. PINA and STRING are comprehensive web sources providing information about protein‒protein interactions, including physical and functional associations between proteins^48,49^.

### Statistical analysis

The results are expressed as the mean ± standard error of the mean (SEM) or standard deviation (SD) of >3 independent samples, as calculated using Microsoft Excel 2010 and the SPSS statistical software package. We chose the minimum sample size required for an acceptable probability of achieving a statistical criterion of interest (e.g., statistical significance or maximum interval width) for the proposed study. The criteria for excluding samples or data, including failure, met our quality control standards for conditions such as insufficient sample volume, unacceptable levels of contaminants, and poor histological quality. Group data for all assays were compared using two-tailed or unpaired Student’s *t test*, and statistical tests were two-sided. The nonparametric statistical tests used as appropriate were the Mann‒Whitney and chi-square tests. Statistical significance was set at *P* < 0.05.

## Results

### SIRT1 expression is aberrantly increased in NSCLC harboring *KRAS*^*Mut*^

**Fig. 1:**
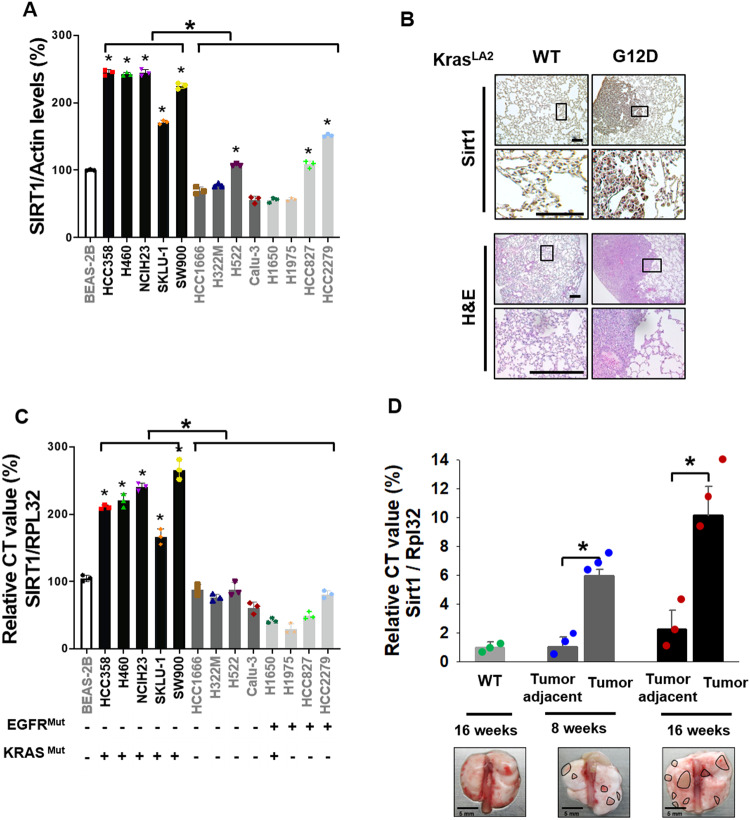
SIRT1 is aberrantly upregulated in KRAS^Mut^ NSCLC cell lines and tumors. **A** SIRT1 protein expression was measured in one noncancerous lung epithelial cell line (BEAS-2B), five KRAS^Mut^ cell lines (H358, H460, NCIH23, SKLU-1, and SW960), a KRAS^Mut^/EGFR^Mut^ cell line (H1650), five KRAS^WT^/EGFR^WT^ cell lines (HCC1666, H322M, H522, Calu-3, and H1650), and three EGFR^Mut^ cell lines (H1975, HCC827, and HCC2279). The area density of each band was measured with ImageJ software. The data were normalized to actin and are presented as the ratio with respect to the area density of actin. The data are plotted relative to the values obtained in the BEAS-2B cell line with *SIRT1* expression. Student’s *t* test, mean ± SD; *n* = 3; *p < 0.05. **B** Representative immunohistochemical staining for SIRT1 and H&E staining in Kras^LA2_WT^ and Kras^LA2_G12D^ Tg mouse lungs at 16 weeks of age. Representative images are shown. Scale bar, 100 μm. The high-magnification images correspond to the areas marked by the black box. **C** SIRT1 mRNA expression was measured by quantitative real-time PCR in the same cell lines shown in Fig. 1A. *RPL32* was used as an internal control and for normalization. Student’s *t* test, mean ± SD; *n* = 3; *p < 0.05. **D** The mRNA expression of Sirt1 was measured by RT‒qPCR in cancerous and adjacent noncancerous lung tissues of Kras^LA2_G12D^ Tg mice. *Rpl32* was used as an internal control and for normalization. Student’s *t* test, mean ± SEM; *n* = 3; *p < 0.05.

SIRT1 expression is associated with poor prognosis in NSCLC patients and may help to identify NSCLC patients with a high risk of recurrence who could benefit from adjuvant therapy after resection^50^. Therefore, we first evaluated the expression of SIRT1 in a panel of human NSCLC cells and nontumorigenic lung epithelial cells using western blotting (Fig. 1A and Supplementary Fig. 1) and quantitative real-time PCR (Fig. 1C). Notably, the protein and mRNA levels of SIRT1 were significantly higher in *KRAS*^*Mut*^ cell lines than in normal lung epithelial cells and in *KRAS*^*Mut*^-negative and *EGFR*^*Mut*^-positive cell lines. To confirm the previous results of SIRT1 expression analysis in cell lines, the protein level of SIRT1 was evaluated with immunohistochemical staining in lung tissues of *Kras*^*LA2_WT*^ and *Kras*^*LA2_G12D*^ mice in the spontaneous tumorigenesis model at 16 weeks. The protein levels of Sirt1 in lung tumors from *Kras*^*LA2_G12D*^ mice were much higher than those in the corresponding tumor-adjacent normal tissues and in normal control lung tissue from *Kras*^*LA2_WT*^ mice (Fig. 1B). In *Kras*^*LA2_G12D*^ mice, lung tumors began to develop eight weeks after birth. Tumors in *Kras*^*LA2_G12D*^ mice were monitored until 16 weeks and found to develop in a time-dependent manner, but tumors were not developed in *Kras*^*LA_ WT*^ mice (Supplementary Fig. 2). In addition, the mRNA expression of SIRT1 was significantly higher in *Kras*^*LA2_G12D*^ lung tumors than in the corresponding tumor-adjacent tissues and *Kras*^*LA2_WT*^ mouse lung tissues in the mice referenced in Fig. 1B at 8 and 16 weeks (Fig. 1D). These results suggest that KRAS^Mut^ upregulates the mRNA and protein expression of SIRT1 in KRAS^Mut^ lung cancer cells, indicating that there might be a positive correlation between KRAS^Mut^ and SIRT1 expression.

### KRAS^Mut^-mediated induction of SIRT1 expression is mediated by c-Myc

**Fig. 2:**
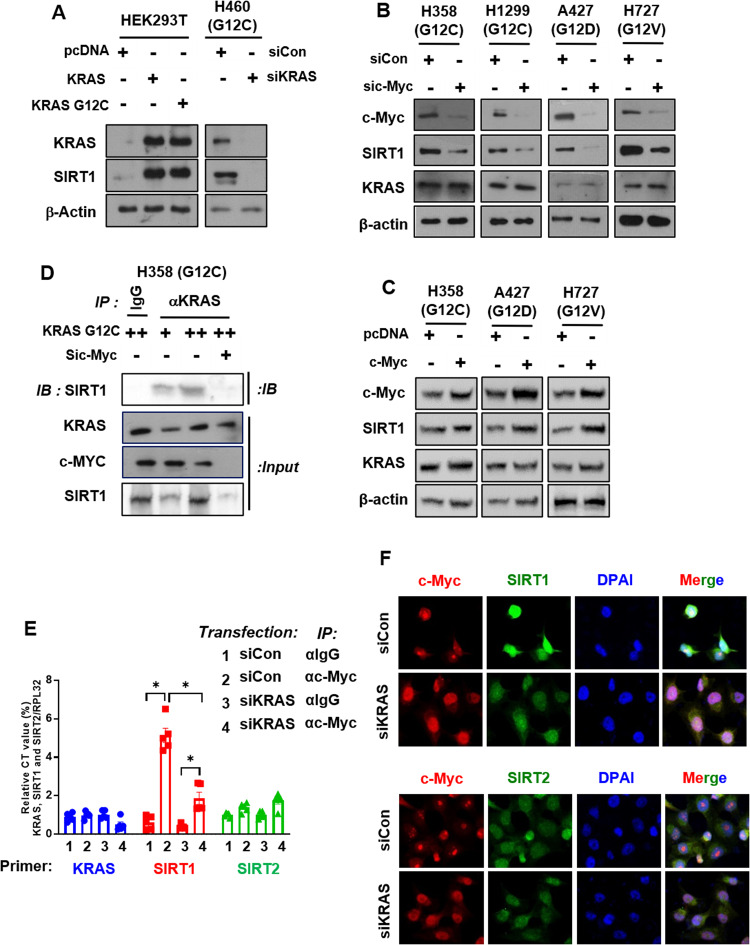
SIRT1 upregulation is mediated by c-Myc downstream of KRAS. **A** HEK293T cells were transfected with *pcDNA* and *KRAS*^*G12C*^ plasmids (2 μg). H460 cells were transfected with siCon and siKRAS (80 nM). The cells were harvested with lysis buffer and subjected to western blotting. **B**, **C** KRAS^Mut^ cells (H358, NCIH23, SKLU-1, SW900, A427, H727), KRAS^WT^ cells, and KRAS^G12C^ cells (H1299^G12C^) were transfected with siCon, c-Myc specific siRNA (80 nM), *pcDNA*, or *c-Myc* plasmid (2 μg) for 48 h, and the levels of the KRAS downstream effectors c-Myc and SIRT1 were measured. **D** H358 cells were transfected with *KRAS*^*G12C*^ and siCon or sic-Myc, and cell extracts were immunoprecipitated with an anti-KRAS antibody and immunoblotted with anti-SIRT1, anti-c-Myc, and anti-KRAS antibodies. **E** Chromatin immunoprecipitation-qPCR analysis of KRAS, SIRT1, and SIRT2 was performed in H358 cells transfected with siCon or siKRAS (80 nM) for 48 h and then immunoprecipitated using an anti-c-Myc antibody or mouse IgG as a negative control. The relative enrichment was calculated by normalizing the qPCR signals. The data are plotted as the mean values determined from at least two independent chromatin immunoprecipitation assays and three independent amplification reactions. Student’s *t* test, mean ± SD; *n* = 6; *p < 0.05. **F** H358 cells were transfected with siCon and siKRAS (80 nM) for 48 h and then fixed after 4 h. *c-Myc* expression was detected with an RFP emission filter, and SIRT1 expression was detected with a GFP emission filter.

To examine how SIRT1 expression is induced by KRAS^Mut^, we performed a gain- and loss-of-function study. First, KRAS^Mut^ cell lines such as H358^G12C^, H1299 (with stable G12C expression), A427^G12D^, and H727^G12V^ were transfected with each of two specific siRNAs for KRAS^Mut^ knockdown (K/D). Both siRNAs dramatically decreased KRAS expression, which also decreased the protein levels of pERK, c-Myc, and SIRT1 but not sirtuin2 (SIRT2). (Supplementary Fig. 3). In contrast, KRAS^G12C^, KRAS^G12D^, and KRAS^G12V^ plasmids were transfected individually into the KRAS^Mut^ lung cancer cell lines H358, A427, and H727. The mRNA expression of SIRT1 but not that of SIRT2 was increased by transfection of the KRAS^Mut^ plasmids (Supplementary Fig. 4a). Transfection of these KRAS^Mut^ plasmids also increased the protein levels of pERK, c-Myc, and SIRT1 but not SIRT2 (Supplementary Fig. 4b). In addition, transfection of the *KRAS*^*WT*^ and *KRAS*^*G12C*^ plasmids increased SIRT1 expression in HEK293T cells, whereas si*KRAS* (#1 siRNA in Supplementary Fig. 3) transfection decreased SIRT1 expression in H460^G12C^ cells (Fig. 2A). SIRT1 expression is regulated by the ERK/MAPK signaling pathway, which is activated by oncogenic *KRAS*^*WT*^ or *KRAS*^*Mut*^ expression. Previous research has shown that the expression of the transcription factor c-Myc is regulated by the MAPK pathway and that c-Myc functions by recognizing and binding to an E-box sequence to drive the transcription of numerous target genes, including SIRT1^51–53^. c-Myc K/D decreased SIRT1 expression independent of KRAS expression in *KRAS*^*Mut*^ cells (Fig. 2B). In contrast, c-Myc plasmid expression increased SIRT1 expression in only KRAS^Mut^ cells (Fig. 2C). To reexamine whether *KRAS*^*Mut*^-induced SIRT1 affects KRAS^Mut^ and determine whether c-Myc plays a downstream role in SIRT1 expression, immunoprecipitation with an anti-KRAS antibody was performed in H358 cells. *KRAS*^*G12C*^ plasmid expression increased SIRT1 expression in a manner dependent on the transfected plasmid concentration, and the rebinding of this additional SIRT1 to KRAS^Mut^ depended on the SIRT1 expression level; this effect was abolished by c-MYC K/D (Fig. 2D). These results demonstrate that KRAS^Mut^ upregulates SIRT1 through the ERK/MAPK-c-Myc pathway but does not upregulate SIRT2 in KRAS^Mut^ lung cancer cells. KRAS^Mut^-induced SIRT1 also rebinds KRAS^Mut^.

The class III histone deacetylase SIRT2 is upregulated by N-Myc in neuroblastoma cells and by c-Myc in pancreatic cancer cells, and SIRT2 enhances N-Myc and c-Myc protein stability and promotes cancer cell proliferation^54^. Therefore, we investigated whether the KRAS^Mut^-ERK/MAPK-c-Myc axis increases SIRT1 or SIRT2 expression in KRAS^Mut^ lung cancer cells. In H358 KRAS^Mut^ cells, KRAS^Mut^-induced c-Myc transcription significantly increased the mRNA and protein levels of SIRT1, which were robustly reduced by KRAS K/D, but did not affect the mRNA and protein expression of SIRT2, as indicated by the ChIP assay (Fig. 2E) and immunofluorescence staining (Fig. 2F). In addition, it has been reported that histone deacetylase 6 (HDAC6) and SIRT2 deacetylate KRAS^Mut^ at lysine 104 to increase the survival of *KRAS*^*Mut*^ pancreatic cancer cells^55^. To confirm whether SIRT2 or HDAC6 regulates the acetylation and activity of the KRAS^Mut^ protein in lung cancer cells, immunoprecipitation with an anti-KRAS antibody was performed in H358 cells. Although both SIRT2 and HDAC6 bound the KRAS^Mut^ protein, neither greatly affected the acetylation or activity of the KRAS^Mut^ protein in lung cancer cells (Supplementary Fig. 5).

In addition, SIRT1 interacts with and deacetylates c-Myc and increases c-Myc stability and phosphorylation at Ser62 in acute myeloid leukemia^56^. Therefore, to examine whether KRAS^Mut^-induced SIRT1 regulates KRAS^Mut^ or c-Myc, the highest expression level of SIRT1 was determined after expression of *KRAS*^*G12C*^. The increase in the SIRT1 level peaked 3.5 days after *KRAS*^*G12C*^ transfection (Supplementary Fig. 6a). Thus, the effect of SIRT1 on KRAS^G12C^ and c-Myc activity was evaluated at this timepoint. KRAS^G12C^-induced SIRT1 affects KRAS^G12C^ activity but not c-Myc activity. This is because SIRT1 increased the level of KRAS^G12C^ activity to a greater degree than it increased c-Myc phosphorylation (Supplementary Fig. 6b). In addition, to identify whether SIRT1 deacetylates the Raf-MEK-c-Myc complex, immunoprecipitation was performed with an anti-SIRT1 antibody. SIRT1 did not form any complex with Raf-MEK-c-Myc but did interact with the KRAS^Mut^ protein (Supplementary Fig. 6c). Based on these results, KRAS^Mut^-induced SIRT1 forms a robust positive feedback loop with KRAS^Mut^ via the Raf-MEK-c-Myc pathway by increasing the level of SIRT1 mRNA, which maintains KRAS^Mut^ activation.

### SIRT1 binds, deacetylates, and regulates the activity of KRAS^Mut^

**Fig. 3:**
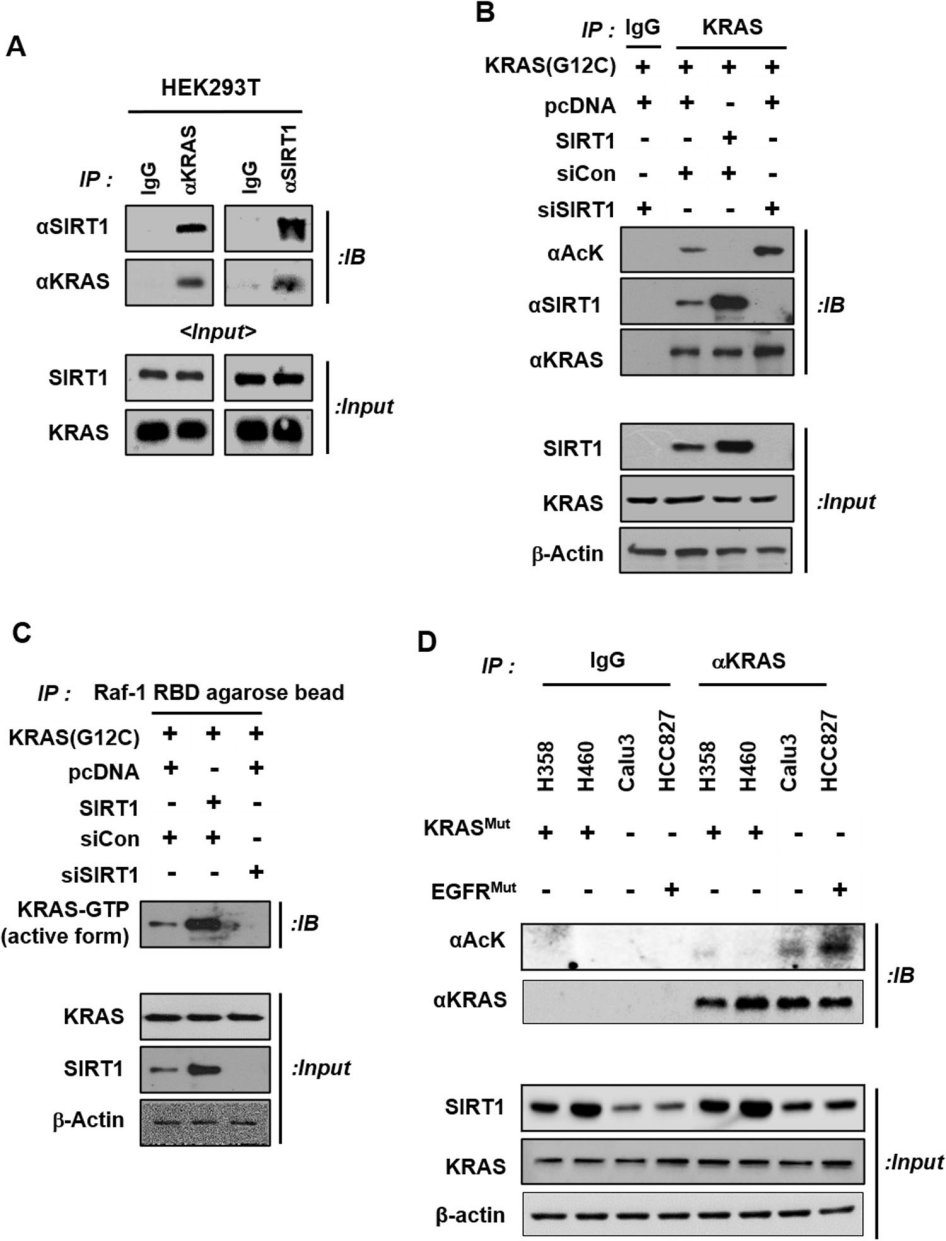
KRAS^Mut^-induced SIRT1 rebinds to KRAS^Mut^ and increases KRAS activity via deacetylation. **A** HEK293T cells were transfected with *KRAS*^*G12C*^ and *SIRT1* plasmids (4 μg), and cell extracts were immunoprecipitated with anti-KRAS and anti-SIRT1 antibodies and immunoblotted with the reciprocal antibody. **B**, **C** Plasmids (*pcDNA*, *KRAS*^*G12C*^, and *SIRT1* each 4 μg) and siRNAs (siCon and siSIRT1, each 80 nM) were transfected into HEK293T cells. Cell extracts were immunoprecipitated with an anti-KRAS antibody and Raf-1 agarose beads and analyzed using anti-acetylated lysine, anti-SIRT1, anti-KRAS, and anti-KRAS-GTP antibodies. **D** Normal lung epithelial cell, fibroblast, and KRAS Mut cell lysates were immunoprecipitated with an anti-KRAS antibody and immunoblotted with anti-acetyl-lysine and anti-KRAS antibodies.

**Fig. 4:**
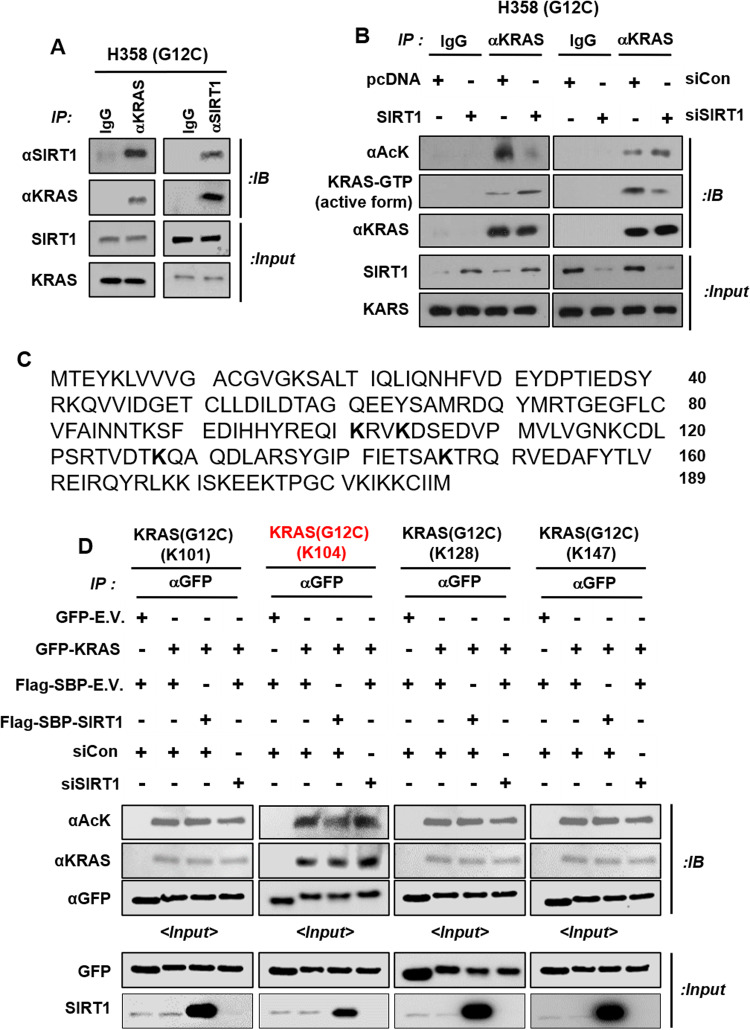
SIRT1 deacetylates lysine 104 of KRAS^G12C^. **A** H358 cell extracts were immunoprecipitated with anti-KRAS and anti-SIRT1 antibodies and immunoblotted with anti-KRAS and anti-SIRT1 antibodies. **B** H358 cells were transfected with *pcDNA* and *SIRT1* (4 μg), siCon, and siSIRT1 (80 nM) and then immunoblotted with anti-acetylated lysine, anti-KRAS-GTP, and anti-KRAS antibodies. **C** The amino acid sequence of KRAS^G12C^ is shown. Lysine acetylation residues are marked as **K** in bold font. **D** HEK293T cells were transfected with *GFP-E.V*., *GFP-KRAS*^*G12C*^ (with three of the four lysine acetylation residues [K101, K104, K128, and K147] sequentially mutated to arginine), *Flag-SBP-E.V*., *Flag-SBP-SIRT1*, siCon, and/or siSIRT1 and incubated for 48 h. Protein lysates were subjected to immunoprecipitation with an anti-GFP antibody and then immunoblotted using anti-acetylated lysine, anti-KRAS, and anti-GFP antibodies.

To investigate how KRAS^Mut^-c-Myc axis-induced SRIT1 regulates KRAS^Mut^ activity, coimmunoprecipitation was performed to investigate the interaction between KRAS^G12C^ and SIRT1. The interaction between KRAS^G12C^ and SIRT1 was examined in HEK293T cells with *KRAS*^*G12C*^ and *SIRT1* plasmid overexpression and in H358 cells with endogenous levels of *KRAS*^*G12C*^ and *SIRT1* expression. KRAS^G12C^ coprecipitated with SIRT1, and this interaction was also crosschecked by reversing the antibodies used for immunoprecipitation and immunoblotting (Figs. 3A and 4A). Several studies have reported that the activity of TRP53, RELA/NF-κBp65, HIF-1α, and PML proteins is decreased by deacetylation of SIRT1 and that the activity of XRCC6/Ku70 and LKB1 is increased by SIRT1^57–62^ (Supplementary Table 1). In addition, the deacetylation sites of KRAS^WT^ were explored using the ASEB method, which revealed that KRAS could be deacetylated at lysine 104 by SIRT1 (*p* value 0.5089, top 39%) and by HDAC1/HDAC2/HDAC3 (*p* value 0.4941, top 41%) (Supplementary Table 2). A lower percentage and a lower *p* value indicate a higher probability of deacetylation by the selected enzyme, and SIRT1 was further evaluated in terms of the shortest pathways via the PINA and STRING databases based on the ASEB method. PINA and the STRING analysis identified the protein networks with the shortest pathways between SIRT1 and KRAS (Supplementary Fig. 7). PINA identified interactive relationships involving a direct interaction of KRAS with SIRT1 (Supplementary Fig. 7a), and STRING analysis revealed that the shortest pathways between SIRT1 and KRAS were through cellular tumor antigen p53 (TP53), polyubiquitin-C (UBC), and the serine/threonine-protein kinase STK11 (Supplementary Fig. 7b).

KRAS acetylation reduces GEF-mediated nucleotide exchange, which decreases GTP reloading^16^. Therefore, to examine whether KRAS^Mut^ activity is increased by SIRT1, we measured the acetylation of KRAS^WT^ and KRAS^G12C^ after *SIRT1* overexpression or K/D. As expected, *SIRT1* expression was increased by expression of KRAS^G12C^ compared to KRAS^WT^. KRAS^Mut^-induced SIRT1 deacetylated KRAS^Mut^ and increased KRAS^Mut^ activity to a greater degree than KRAS^WT^, and this effect was attenuated by SIRT1 K/D in HEK293T cells (Fig. 3B, C). In addition, when the endogenous acetylation levels of KRAS^WT^ and KRAS^Mut^ were compared among normal lung epithelial cells, fibroblasts, KRAS^Mut^ cells, and EGFR^Mut^-positive cells, the acetylation levels in KRAS^Mut^ cell lines were the lowest among normal lung epithelial cells, fibroblasts, and EGFR^Mut^-positive or EGFR^Mut^-negative cells expressing KRAS^WT^, because KRAS^Mut^ activity depends on the SIRT1 expression level (Fig. 3D and Supplementary Fig. 8). To examine whether the changes in the acetylation and activity of KRAS^Mut^ induced by SIRT1 expression or K/D depend on the type of KRAS mutation, immunoprecipitation with an anti-KRAS antibody was performed in H358^G12C^, A427^G12D^, and H727^G12V^ cell lines. In accordance with previous results, the acetylation of KRAS^Mut^ was decreased by SIRT1 expression, and this effect increased the activity of KRAS^Mut^. In contrast, SIRT1 K/D increased KRAS^Mut^ acetylation and diminished KRAS^Mut^ activity in these three KRAS^Mut^ cell lines (Fig. 4B and Supplementary Fig. 9). SIRT1 K/D also increased KRAS^Mut^ acetylation and reduced KRAS^Mut^ activity in the H1299^G12C^ stable cell line. (Supplementary Fig. 10c). This stable cell line was confirmed by western blotting, GFP staining, and a cell proliferation assay (Supplementary Fig. 10a, b). In addition, to confirm whether SIRT1 acetylation affects KRAS^Mut^ activity, KRAS^G12C^ activity was measured after expression of SIRT1^WT^ and SIRT1^H363Y^ (a catalytically inactive SIRT1 mutant). Expression of SIRT1^H363Y^ increased the acetylation level and decreased the activity of KRAS^G12C^ compared with those in the groups transfected with pcDNA or the SIRT1 plasmid (Supplementary Fig. 11). It has been reported that KRAS^WT^ can be acetylated at four lysine residues: K101, K104, K128, and K147^63^ (Fig. 4C). To identify the lysine residue of KRAS^Mut^ that is deacetylated by SIRT1, three of the four lysine acetylation sites in KRAS^G12C^ were mutated to arginine. Deacetylation of only K104 was decreased by SIRT1 overexpression, and this decrease was reversed by SIRT1 K/D (Fig. 4D).

To confirm whether KRAS^G12C_K104^ plays a role in regulating KRAS^Mut^ activity, we generated an H1299 KRAS^G12C_K104R^ stable cell line. KRAS^Mut^ activity in H1299 KRAS^G12C_K104R^ cells was lower than that in H1299 KRAS^G12C_K104^ cells and was not changed by SIRT1 O/E or K/D (Supplementary Fig. 12a). When we investigated the levels of the KRAS^Mut^ downstream signaling mediators pAkt and pERK, we found that the phosphorylation of Akt and ERK was increased in H1299 KRAS^G12C_K104^ cells but decreased in H1299 KRAS^G12C_K104R^ cells compared with H1299 KRAS^WT^ cells (Supplementary Fig. 12b). Based on the previous results, H1299 KRAS^G12C_K104R^ cells formed more colonies than H1299 KRAS^WT^ and H1299 KRAS^G12C_K104R^ cells. H1299 KRAS^G12C_K104R^ cells formed significantly fewer colonies than H1299 KRAS^G12C_K104^ cells (Supplementary Fig. 12c). These results are in accordance with previous reports that KRAS^K104R^ suppresses GEF activity^16^ and impairs SOS-mediated nucleotide exchange^64^. Overall, these findings indicate that the K104 lysine residue in KRAS^Mut^ is deacetylated by SIRT1, which increases KRAS^Mut^ activity via stable reloading of GTP.

### SIRT1 reverses p300-mediated lysine 104 acetylation of KRAS^Mut^

**Fig. 5:**
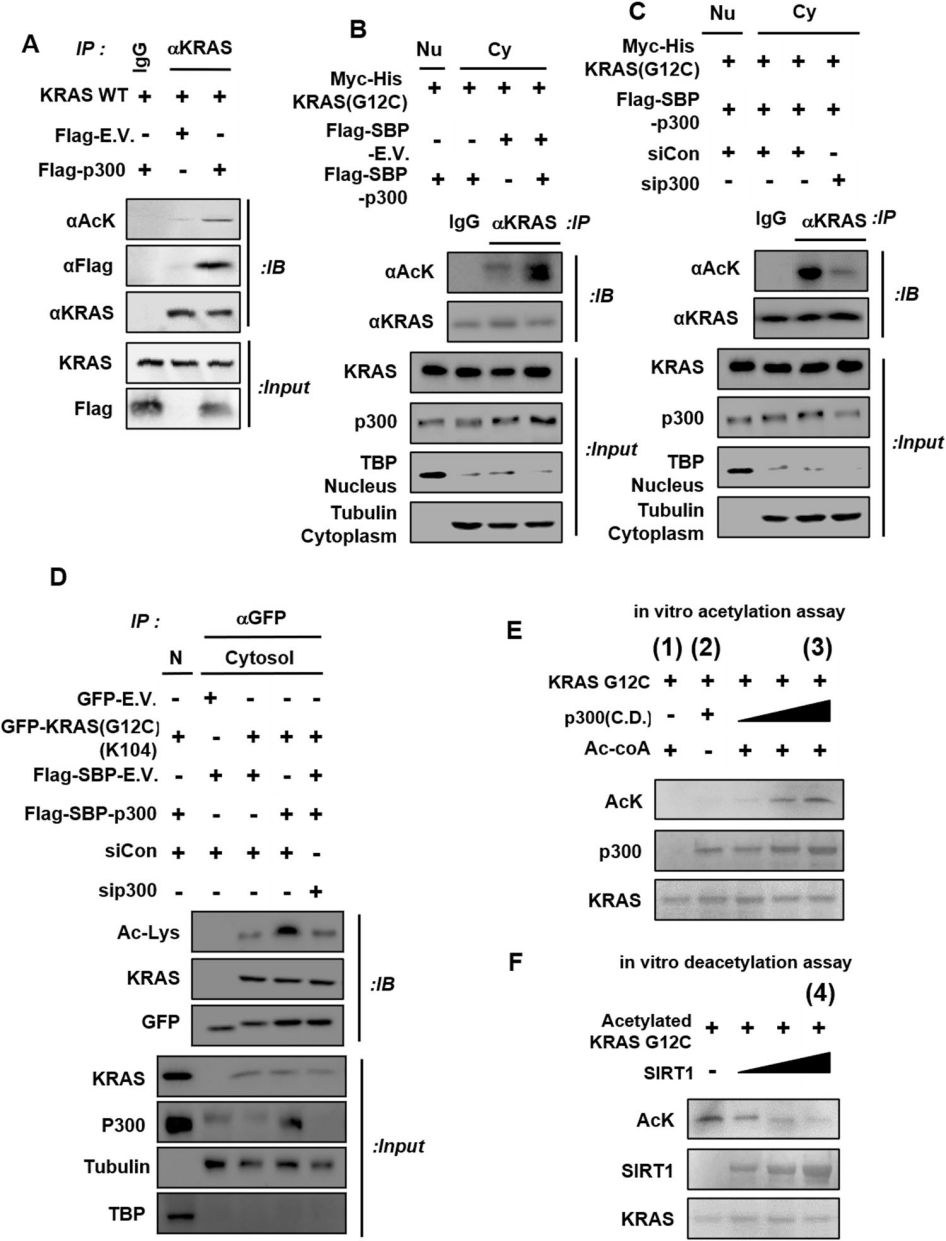
KRAS^Mut^ lysine 104 is acetylated by the p300 acetyltransferase. **A** HEK293T cells were transfected with *KRAS*^*WT*^, *Flag-E.V*., and *Flag-p300* plasmids. Cell lysates were immunoprecipitated with IgG and an anti-KRAS antibody and then immunoblotted with anti-acetyl-lysine, anti-Flag, and anti-KRAS antibodies. **B**, **C** *Myc-His-KRAS*^*G12C*^, *Flag-SBP-E.V., Flag-SBP-p300*, siCon, and/or sip300 were overexpressed in HEK293T cells; after 48 h, cell lysates were separated into nuclear and cytoplasmic fractions, which were subjected to immunoprecipitation with an anti-KRAS antibody and immunoblotting with anti-acetylated lysine and anti-KRAS antibodies. TATA-binding protein was used as a nuclear marker, and tubulin was used as a cytoplasmic marker to verify that nuclear separation was successful. **D** HEK293T cells were transfected with *GFP-E.V., GFP-KRAS* (all lysine residues between a.a. 101 and 147 except for K104 mutated to arginine), *Flag-SBP-E.V*., *Flag-SBP-p300*, siCon, and/or sip300. Protein lysates were immunoprecipitated with an anti-GFP antibody and then immunoblotted with anti-acetylated lysine, anti-KRAS, and anti-GFP antibodies. **E** The recombinant KRAS^G12C^ protein and the p300 C.D. (catalytic domain, aa 1284–1674, 45.1 kDa) were incubated in the acetylation assay reaction mixture at 32 °C for 4 h, and lysine-acetylated peptides were then identified using an anti-acetyl-lysine antibody. **F** Lysine-acetylated KRAS^G12C^ peptides were also incubated with recombinant SIRT1 protein in the deacetylation assay reaction mixture for 4 h. Deacetylation was quantified using an anti-acetyl-lysine antibody.

We investigated which acetylation enzymes are involved in KRAS^WT^ acetylation. First, KRAS^WT^ acetylation was measured after overexpression of several acetyltransferase candidates, such as p300, TIP60, PCAF, and GCN5 (Fig. 5A and Supplementary Fig. 13). Among acetyltransferases, only p300 bound to KRAS^WT^, and KRAS^WT^ acetylation was positively regulated. Next, to investigate whether the p300 acetyltransferase is involved in KRAS^Mut^ acetylation, KRAS^G12C^ acetylation was measured after overexpression of the KRAS^G12C^ plasmid, p300 plasmid, and/or sip300 in HEK293T cells. Overexpression of p300 increased KRAS^G12C^ acetylation (Fig. 5B), and p300 K/D decreased KRAS^G12C^ acetylation (Fig. 5C) compared to KRAS^G12C^ acetylation in the control group. The p300 acetyltransferase is known as a histone acetyltransferase, whereas the acetylation of KRAS^Mut^ occurs in the cytosol. Moreover, to confirm whether p300 is responsible for the acetylation of KRAS^Mut^ in the cytosol, all immunoprecipitation reactions were performed with cytosol samples after nuclear extraction. Furthermore, to examine whether K104, the site deacetylated by SIRT1, is acetylated by p300, the plasmids containing mutations in three lysine acetylation sites in KRAS^G12C^ used in the experiments referenced in Fig. 4D were transfected with the p300 plasmid or sip300 into HEK293T cells. In accordance with previous results, the site of p300-mediated acetylation in KRAS^G12C^ was K104, which was deacetylated by SIRT1 (Fig. 5D and Supplementary Fig. 14). In addition, to confirm whether the three KRAS^Mut^ types, G12C, G12D, and G12V, are acetylated by p300, KRAS^Mut^ acetylation and activity were measured after the expression of p300 and sip300 in H358^G12C^, A427^G12D^, and H727^G12V^ cells. The acetylation and activity of all three KRAS^Mut^ types were regulated by overexpression and K/D of p300 (Supplementary Fig. 15).

To examine whether SIRT1 directly reverses p300-mediated K104 acetylation, we performed in vitro acetylation and deacetylation assays. The recombinant KRAS^G12C^ peptide was acetylated by the recombinant p300 (C.D., catalytic domain) peptide in a dose-dependent manner and was deacetylated by the recombinant SIRT1 peptide in an NAD^+^-dependent manner (Fig. 5E, F). To confirm the results of previous in vitro acetylation and deacetylation assays, the KRAS^G12C^ peptide mass in samples (1), (2), (3), and (4) described in Fig. 5E, F was measured by LC‒MS/MS. Only sample (3) in Fig. 5E, containing both KRAS^G12C^ and p300 was found to exhibit K104 acetylation, whereas sample (4) in Fig. 5F, containing the SIRT1 peptide, did not, as determined by the MS/MS data (Supplementary Fig. 16). These results suggest that SIRT1 and p300 dominantly regulate the K104 acetylation of KRAS^Mut^.

### SIRT1 deletion increases apoptosis and improves the efficacy of chemotherapy in *Kras*^*Mut*^-driven lung cancer

**Fig. 6:**
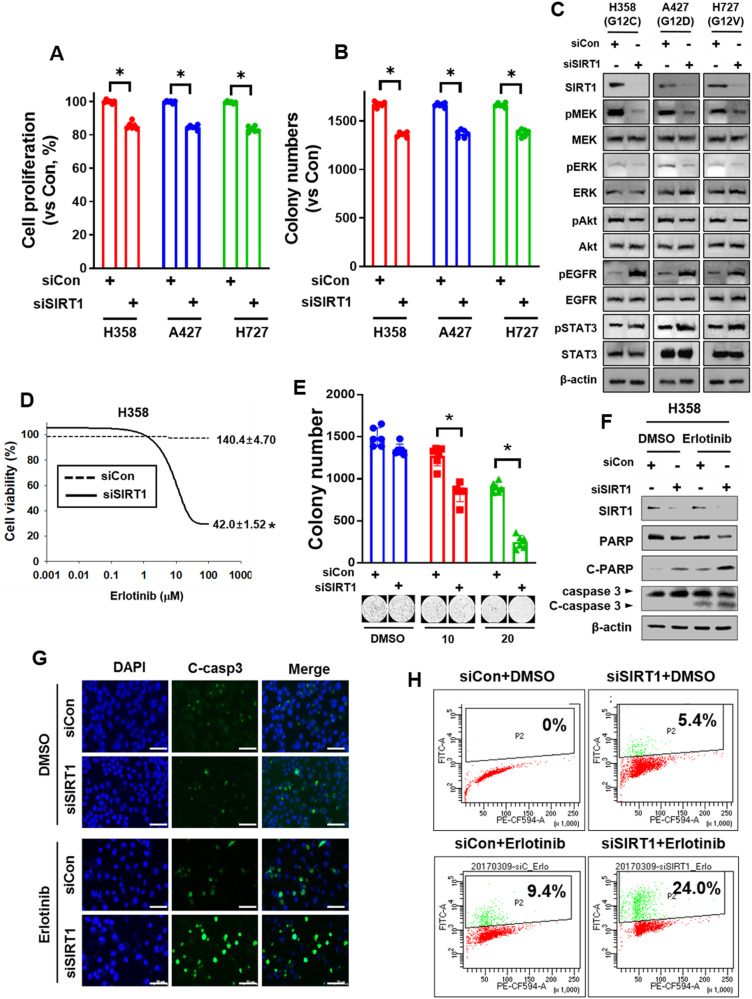
SIRT1 inactivation increases apoptosis and therapeutic efficacy in KRAS^Mut^ cells. **A**, **B** and **D**, **E** H358 cells were transfected with siCon or siSIRT1 (80 nM) and then seeded into 96-well plates for a growth assay **A**, **D** and into 12-well plates on agarose gel for a colony formation assay **B**, **E**. **A**, **B** without erlotinib; **D**, **E** dose-dependent treatment with erlotinib. Student’s *t* test, mean ± SD; *n* = 6; *p < 0.05. **C** H358, A427, and H727 cells were transfected with siCon or siSIRT1 (80 nM) and then immunoblotted with anti-SIRT1, anti-pMEK, anti-MEK, anti-pERK, anti-ERK, anti-pAkt, anti-Akt, anti-pEGFR, anti-EGFR, anti-pSTAT3, anti-STAT3, and anti β-actin antibodies. **F**–**H** H358 cells were transfected with siCon or siSIRT1 (80 nM) and treated with erlotinib (10 μM). **F** Cell lysates were immunoblotted with anti-PARP and anti-caspase-3 antibodies. **G** Cells treated with the drugs were subjected to immunofluorescence staining and **H** analysis of DNA damage using the APO-BrdU TUNEL assay.

As mentioned above, KRAS^Mut^-induced SIRT1 rebinds to KRAS^Mut^ and deacetylates it, which increases KRAS^Mut^ activity. However, SIRT1 K/D decreased KRAS^Mut^ activity and the downstream signaling of pERK and pAkt. Therefore, to examine whether SIRT1 K/D affects *KRAS*^*Mut*^-driven proliferation in lung cancer cell lines, we performed cell viability assays and colony formation assays in H358, A427, and H727 cells after SIRT1 K/D. Even though the proliferation rates and colony numbers of cells transfected with siSIRT1 were significantly decreased compared with those in the siCon group, the reductions were less than 20% (Fig. 6A, B). These results indicate that the inactivation of *KRAS*^*Mut*^-induced SIRT1 is not sufficient to inhibit KRAS^Mut^ lung cancer cell proliferation.

Therefore, we investigated why SIRT1 K/D is insufficient to exterminate KRAS^Mut^ lung cancer proliferation. SIRT1 K/D decreased the phosphorylation of MEK and ERK. Unlike the levels of pMEK and pERK, the pAkt level was not reduced because of SIRT1 K/D, whereas the phosphorylation of EGFR and signal transducer and activator of transcription 3 (STAT3) was increased (Fig. 6C). In previous reports, Akt activation was found to increase the activity of A Disintegrin and Metalloproteinase 17 (ADAM17), which increases the shedding of heparin-binding EGF (HB-EGF) and amphiregulin (AREG) from the cancer cell surface, leading to EGFR activation^65,66^. To confirm whether SIRT1 K/D-induced AKT phosphorylation increases the activity of ADAM 17, EGFR, and STAT3, H358, A427, and H727 cells were transfected with siCon and siSIRT1 and treated with LY294002 (a PI3K inhibitor). The induction of AKT, ADAM17, EGFR, and STAT3 activity by SIRT1 K/D was attenuated by Akt inhibition (Supplementary Fig. 17a). Furthermore, the increase in ADAM17 activity induced by SIRT1 K/D increased the levels of shed HB-EGF and AREG in the supernatant of all three cell lines, and this increase was completely abrogated by transfection of an ADAM17-specific siRNA (Supplementary Fig. 17c). The K/D efficiency of siADAM17 was confirmed by western blotting (Supplementary Fig. 17b). These results suggest that MEK/ERK signaling is positively regulated by KRAS^Mut^, whereas EGFR/STAT3 signaling is negatively regulated by KRAS^Mut^ to compensate for the reduction in KRAS^Mut^ activity by enhancing the shedding of HB-EGF and AREG.

To examine whether treatment with EGFR TKIs (tyrosine kinase inhibitors) to reduce EGFR phosphorylation induced by SIRT1 K/D exerts synergistic anticancer effects, cell viability and colony formation assays were performed with different concentrations of erlotinib under SIRT1 K/D conditions in H358^G12C^, NCIH23^G12C^, and H1299^G12C^ stable cell lines. In the cell viability assay, the IC_50_ of erlotinib in all KRAS^Mut^ SIRT1 K/D cells was significantly decreased compared to that in the corresponding control group (Fig. 6D, Supplementary Figs. 18a, 19a). SIRT1 K/D in NCIH23 cells was confirmed by western blotting (Supplementary Fig. 18b). In addition, expression of a catalytically inactive SIRT1 mutant, *SIRT1*^*H363Y*^, also significantly decreased the IC_50_ values of erlotinib and cisplatin in H358 cells (Supplementary Fig. 20a, b) and sensitized cells to the anticancer effect of erlotinib in the colony formation assay. On the other hand, SIRT1 O/E reduced the reduction in the colony number resulting from erlotinib treatment (Supplementary Fig. 21). Furthermore, SIRT1 K/D cells treated with 10 and 20 μM erlotinib showed a dose-dependent decrease in the colony number compared to cells transfected with siCon and treated with or without erlotinib (Fig. 6E and Supplementary Fig. 19b). The results of western blotting, immunofluorescence staining, and the APO-BrdU TUNEL assay demonstrated that SIRT1 K/D in combination with erlotinib increased PARP and caspase-3 cleavage (Fig. 6F–H; Supplementary Fig. 19c–e), indicating that the combination of SIRT1 K/D with chemotherapy drugs or erlotinib could sensitize Kras^Mut^ lung cancer cells to overcome chemoresistance.

**Fig. 7:**
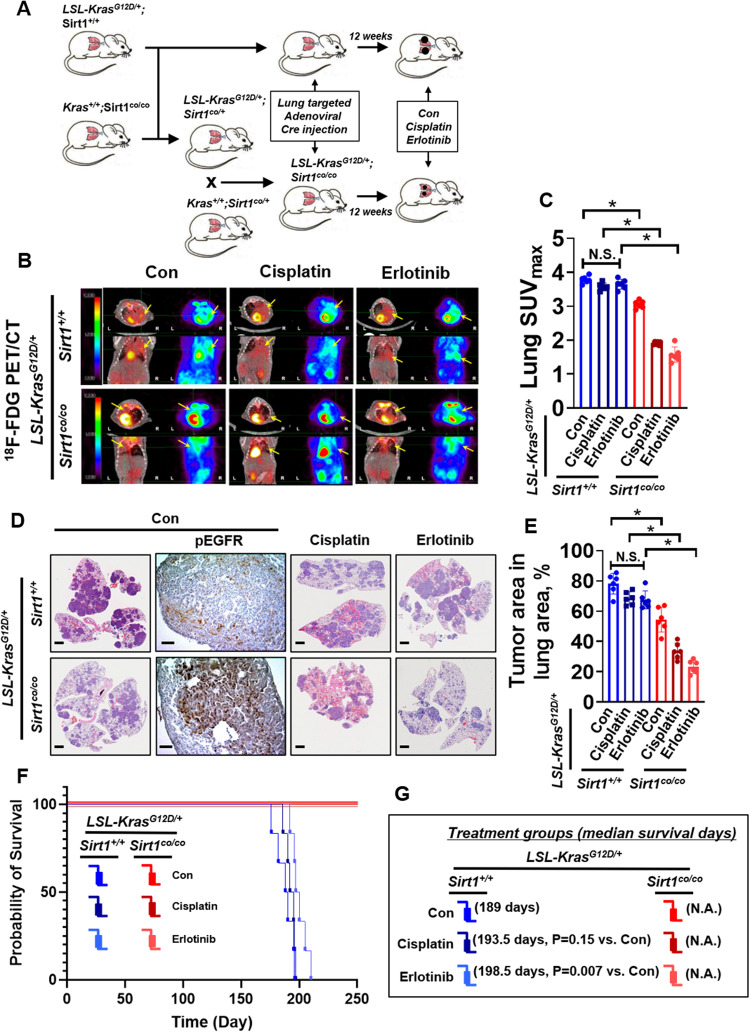
Sirt1 potentiates the anticancer effect of chemotherapy and EGFR TKI treatment against *Kras*^*G12D*^-induced lung tumorigenesis. **A** Schematic of genetic manipulation of LSL-Kras^G12D/+^ and/or Sirt1^co/co^ mice before and after adenoviral Cre administration. **B** PET images of mice between 22 and 23 weeks of age. All images were normalized to the same maximal standard uptake value (SUV_max_) to facilitate the comparison of PET lesions. The yellow arrow indicates the tumor region. Cisplatin (7 mg/kg/1 time/week, i.p.) and erlotinib (15 mg/kg/2 times/week, i.p.) were administered beginning 13 weeks after Ad-cre virus injection. **C** Quantification of the tumor uptake value (SUV_max_) and mean standardized uptake value (SUV_max_) in the lung. Student’s *t* test, mean ± SEM; *n* = 6; *p < 0.05. **D** Representative H&E-stained lung sections from LSL-Kras^G12D/+^;Sirt1^+/+^ and LSL-Kras^G12D/+^;Sirt1^co/co^ mice. The bars represent 800 μm. **E** Average tumor number per lung area in specimens collected from LSL-Kras^G12D/+^;Sirt1^+/+^ (*n* = 6) and LSL-Kras^G12D/+^;Sirt1^co/co^ (*n* = 6) mice between 24 and 27 weeks. Student’s *t* test, mean ± SEM; *n* = 6; *p < 0.05. **F** Survival rates of LSL-Kras^G12D/+^;Sirt1^+/+^ (*n* = 6) and LSL-Kras^G12D/+^;Sirt1^co/co^ (*n* = 6) mice following Cre induction (log-rank test). **G** Median survival times (days) and *P* values were calculated using the log-rank test and Gehan-Breslow-Wilcoxon test on the basis of Student’s *t* test, respectively.

To support the previous in vitro results, we generated a Kras^Mut^-driven lung tumor model (LSL-Kras^G12D/+^) to examine the synergistic anticancer effects of SIRT1 K/D and chemotherapy or EGFR TKI treatment. Infection of LSL-Kras^G12D/+^ mice with an adenovirus encoding Cre recombinase induces the expression of *Kras*^*G12D*^ in the lung epithelium and the formation of lung adenomas that rarely progress to higher-grade tumors^67^. We crossed LSL-Kras^G12D/+^ mice with C57BL6/J mice to generate cohorts with the Sirt1^+/+^ genotype or global Sirt1 knockout (Sirt1^co/co^) (Fig. 7A). The transgenic mouse genotype and SIRT1 K/O (Fig. 7A) were confirmed by PCR genotyping and western blotting with lung samples (Supplementary Figs. 22, 23). As expected from the in vitro results, a reduced tumor burden in Kras^G12D/+^;Sirt1^co/co^ mice treated with cisplatin or erlotinib compared with the age-matched KRAS^G12D/+^;Sirt1^+/+^ mice, mice in each single drug treatment group, and Kras^G12D/+^;Sirt1^co/co^ mice was observed using ^18^F-FDG PET/CT imaging and calculation of lung SUV_max_ values (Fig. 7B, C). We also found that Sirt1 K/O in LSL-Kras^G12D/+^ mice treated with cisplatin or erlotinib resulted in a significantly lower tumor burden than that in KRAS^G12D/+^;Sirt1^+/+^ mice, mice in each single drug treatment group, and Kras^G12D/+^;Sirt1^co/co^ mice, as determined by H&E staining and determination of the tumor number in the lung region (Fig. 7D, E). In accordance with the western blotting data in Fig. 6C, the IHC staining results showed that the phospho-EGFR levels in Kras^G12D/+^;Sirt1^co/co^ mice were higher than those in KRAS^G12D/+^;Sirt1^+/+^ mice (Fig. 7D). In addition to a reduced tumor burden, no deaths occurred in the groups of Sirt1 K/O mice treated with cisplatin or erlotinib, but the median survival time of KRAS^G12D/+^;Sirt1^+/+^ mice and the mice in each single drug treatment group was less than 198.5 days (Fig. 7F, G). Collectively, these data indicate that SIRT1 K/D can sensitize Kras^Mut^ lung cancer cells to chemotherapy or EGFR TKI treatment.

### Synergistic effect of SIRT1 inhibitor treatment and chemotherapy/EGFR TKI treatment in KRAS^Mut^-driven lung cancer

We next verified the synergistic effects of SIRT1 inhibitor treatment combined with cisplatin or EGFR TKI treatment in vitro and in vivo. We assessed whether SIRT1 inhibition could synergize with cisplatin or erlotinib treatment in inducing cell death. Platinum-based chemotherapy (e.g., cisplatin) in combination with EGFR inhibitors, such as erlotinib or gefitinib, is widely used to treat NSCLC. However, lung cancer patients harboring *KRAS* mutations have been shown to be resistant to these anticancer drugs^68^. To investigate whether the SIRT1 inhibitor EX527 showed synergistic anticancer effects when used in combination with cisplatin or erlotinib, H358 cells were treated with EX527 combined with cisplatin or erlotinib. The IC_50_ values in cells treated with EX527 and either cisplatin or erlotinib indicated significant sensitization compared with those in each single treatment group (Supplementary Fig. 24a). In addition, the Chou-Talalay method was used to characterize the synergism of EX527, where the CI provides a quantitative assessment of synergism between drugs^46^. The CI was estimated from the dose-effect data of cisplatin and erlotinib as single agents and in drug combinations. A value of CI < 1 indicated synergism, CI = 1 indicated an additive effect, and CI > 1 indicated antagonism. As shown in the CI plot, both cisplatin and erlotinib had a synergistic effect at low concentrations in combination with EX527 (Supplementary Fig. 24b). This was because EX527 increased the acetylation of KRAS^G12C^, which decreased KRAS^G12C^ activity in H358 cells (Supplementary Fig. 25). When EX527 was combined with cisplatin or erlotinib, the levels of apoptosis markers, cleaved PARP, cleaved caspase 3, PI-Annexin V staining, and Apo-BrdU TUNEL positivity were substantially increased compared with those in each single treatment group or the untreated group, as shown by western blot analysis and immunofluorescence staining (Supplementary Figs. 24de, 26). Therefore, these combined treatments significantly decreased the colony number of KRAS^Mut^ cells compared to that in each single treatment group or the control group (Supplementary Fig. 24c). Collectively, these results indicate that the SIRT1 inhibitor EX527 has potential as an adjuvant therapy for reducing chemoresistance in KRAS^Mut^-driven cancers.

### Combination treatment with a SIRT1 inhibitor and erlotinib exerts synergistic antitumor effects in mouse models of *KRAS*^*G12C*^ orthotopic lung cancer

**Fig. 8:**
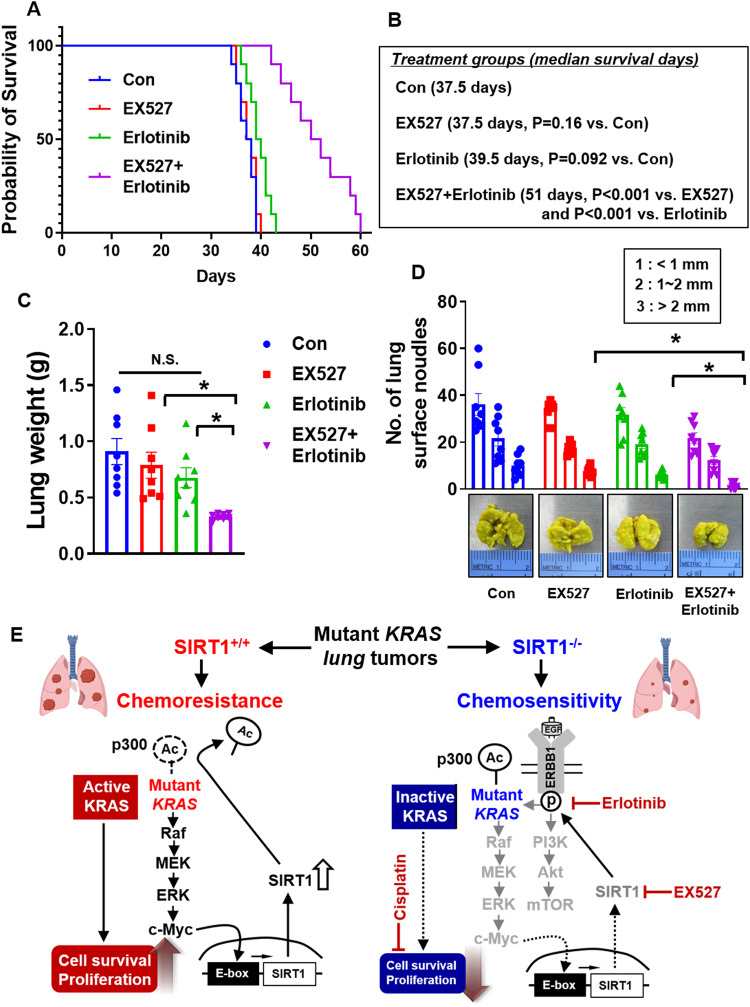
SIRT1 inhibitor treatment is required for the synergistic therapeutic efficacy of EGFR TKIs in the KRAS^G12C^ lung orthotopic tumor model. **A** H358 cells were injected intratracheally into nude mice (1 × 10^6^ cells/mouse). Tumors were allowed to establish for 10 days before the mice were randomized into treatment groups. Survival curves of lung orthotopic tumor-bearing mice. *n* = 10; *p < 0.05. Two-way ANOVA. The mouse survival curves were generated and visualized using the Kaplan‒Meier method. Therapeutic candidate drugs, including EX527 (10 mg/kg/3 times/week, i.p.) and erlotinib (15 mg/kg/2 times/week, i.p.) were administered as specified. Mice were euthanized at the first indication of morbidity, and the lungs were excised and stained with Bouin’s fixative. **B** Median survival times (days) and P values were calculated by the log-rank test and Gehan-Breslow-Wilcoxon test, respectively. *p < 0.001. On the basis of Student’s *t* test. **C** Tumor weights were measured after tumor excision from the mice. Student’s *t* test was performed for statistical analysis, and the values are shown as the means ± SEMs; *n* = 8; *p < 0.05. **D** The colonies formed in the lungs were counted based on colony size: less than 1 mm, between 1 mm and 2 mm, and more than 2 mm. Student’s *t* test was performed for statistical analysis, and the values are shown as the means ± SEMs; *n* = 8; *p < 0.05. **E** Schematic overview of the chemoresistance mechanism of KRAS^Mut^-induced SIRT1 and definition of a rational combination strategy to overcome chemoresistance in KRAS^Mut^ cancers.

To confirm the in vivo benefits of SIRT1 inhibitor and/or erlotinib treatment endowed by KRAS^Mut^, H358 cells were inoculated via intratracheal injection. We found that the number of colonies in the lungs and the lung weight in the combination treatment group were significantly lower than those in each single treatment group and the untreated group and that mice in the combination treatment group harbored the lowest number of grossly visible surface tumors (Fig. 8C, D). The combination therapy resulted in more TUNEL-positive cells than observed in either single treatment group or the control group, which decreased the number of Ki-67-positive cells and ERK phosphorylation. We confirmed the synergistic anticancer effect of EX527 by SIRT1 IHC staining and by evaluation of the tumor burden by H&E staining (Supplementary Fig. 27). The combination treatment of EX527 and erlotinib resulted in a longer median survival time than that in either single treatment group or the untreated group (Fig. 8A, B). These results strongly indicate that EX527-mediated inhibition of SIRT1 in KRAS^G12C^ lung cancer enhanced the efficacy of erlotinib treatment through reduced cell proliferation, enhanced DNA damage, and increased apoptotic cell death, suggesting that the inhibition of SIRT1 in combination with erlotinib treatment constitutes a potential therapeutic strategy for *KRAS*^*Mut*^ lung cancer. A schematic overview summarizing the above findings is shown in Fig. 8E.

## Discussion

RAS is one of the most frequently mutated oncogenes in human cancer and is a pivotal driving factor for tumorigenesis. The three RAS genes encode highly homologous RAS proteins, HRAS, NRAS, and KRAS^43^. KRAS is the isoform most frequently mutated, accounting for approximately 85% of RAS mutations. KRAS-4B is the dominant isoform relative to KRAS-4A in human cancers, and it is present in approximately 90% of pancreatic cancers, 30–40% of colon cancers, and 15–20% of lung cancers, mostly NSCLCs^5^. The KRAS protein functions as a delivery sensor that allows the transduction of signals from the cell surface to the nucleus and promotes several essential cellular processes, such as differentiation, growth, chemotaxis, and apoptosis^6,7^. Although many oncologists have made persistent efforts, effective direct anti-KRAS therapies remain elusive^41,42^. It is very difficult to inhibit KRAS^Mut^ activity by interfering with GTP–GDP binding because of the lack of well-defined hydrophobic pockets on the surface of KRAS^Mut^ proteins, the picomolar affinity of GTP and GDP for KRAS^Mut^ and the high intracellular concentrations of GTP and GDP^69^. Therefore, KRAS^Mut^ proteins are considered challenging targets.

However, the activation of KRAS^Mut^ signaling occurs via a two-step process involving the plasma membrane localization of KRAS and its interaction with effector proteins, processes that are regulated by posttranslational modification (PTM) of KRAS^70^. Blocking these processes can decrease KRAS^Mut^ activation, which interferes with its association with the plasma membrane, regulates its interactions with effectors, and regulates its protein stability. PTMs regulating the association of the KRAS^Mut^ protein with the plasma membrane are prenylation, proteolysis, methylation, palmitoylation, and phosphorylation. Among these PTMs, prenylation occurs in KRAS^Mut^ proteins that contain a CAAX motif as the substrate for farnesyltransferase (FTase) and allows KRAS^Mut^ proteins to have a low affinity for the plasma membrane. Thus, the prenylated KRAS protein translocates to the surface of the endoplasmic reticulum (ER) and interacts with KRAS-converting enzyme 1 (RCE1)^71^. Although prenylation inhibitors have shown anti-KRAS activity, their use is limited by their poor pharmacokinetic properties and high affinity for bone minerals. PTMs affecting KRAS^Mut^ nucleotide exchange from GTP to GDP include nitrosylation, ubiquitination, SUMOylation, and acetylation. In particular, monoubiquitination of KRAS^Mut^ at lysine 147 impairs GAP-mediated GTP hydrolysis, which promotes GTP loading and enhances the affinity of KRAS^Mut^ for downstream effectors such as components of the Raf-MEK-PI3K axis. In addition, mono/diubiquitination of lysine 117 promotes nucleotide exchange, thereby enhancing KRAS^Mut^ activation^71^. Thus, targeting E3 ligases is an alternative method for treating oncogenic KRAS^Mut^-driven cancers. For example, aberrantly activated KRAS^Mut^ undergoes a conformational change, which inhibits Nedd4-1 ubiquitin ligase-mediated polyubiquitination and degradation, resulting in the suppression of *KRAS*^*Mut*^ cancer development^72^. However, this method also has challenges. While some ubiquitin ligases function as tumor suppressors through the degradation of oncogenic KRAS^Mut^, strategies to excessively activate these ubiquitin ligases could be nonspecific for KRAS^Mut^. Therefore, targeting ubiquitination may have results below clinical expectations.

Yang et al. reported lysine 104 acetylation of KRAS. First, K104 acetylation affects the transforming activity of KRAS by interfering with GEF-mediated nucleotide exchange. K104 acetylation is a negative regulatory modification of KRAS^16^. Second, HDAC6- and SIRT2-mediated deacetylation of KRAS^Mut^ lysine 104 increases the survival of *KRAS*^*Mut*^ pancreatic cancer cells^55^. Additionally, lysine 147 was discovered to be a novel substrate for SIRT2-mediated deacetylation, and its acetylation status strongly affects the oncogenic properties of *KRAS*^*Mut*^ pancreatic cancer cells^73^. Notably, we found that the protein and mRNA expression levels of SIRT1 were aberrantly increased in NSCLC cells harboring *KRAS*^*Mut*^ and in *KRAS*^*G12D*^ mouse lung tumors compared with normal lung epithelial cells and with tumor-adjacent normal tissues and *Kras*^*WT*^ mouse lung tissues, respectively (Fig. 1). The KRAS^Mut^-induced increase in SIRT1 expression occurs at the transcription level; thus, we hypothesized that some transcription factors are controlled by the MAPK pathway downstream of KRAS. Consequently, we found that the activity of the KRAS^Mut^-MAPK/ERK downstream transcription factor c-Myc significantly increased the mRNA and protein levels of SIRT1 but not SIRT2 in H358 *KRAS*^*G12C*^ cells (Fig. 2).

However, there was one report stating that the Sirt1 protein level in MEFs from mice overexpressing KrasTg or Sirt1 Tg showed a greater reduction than that in MEFs from mice expressing WT Kras or Sirt1^40^. They also measured SIRT1 levels in seven KRAS mutant cell lines after PI3K inhibitor treatment. Only two cell lines showed reductions in the SIRT1 level similar to those in MEFs from Kras Tg or Sirt1 Tg mice. Interestingly, reduced Sirt1 protein levels were observed in Kras Tg and Sirt1 Tg mice, and the reductions in Sirt1 levels in human Kras mutant cell lines were inconsistent with those in all KRAS mutant cell lines. Then, they compared tumor numbers between Kras^Tg^;Sirt1^WT^ and Kras^Tg^;Sirt1^Tg^ mice^40^. The tumor number in Kras^Tg^;Sirt1^Tg^ mice was less than that in Kras^Tg^;Sirt1^WT^ mice for carcinoma tumors but not for adenoma tumors. In addition, there was no significant difference in tumor growth between Kras^Tg^;Sirt1^Tg^ mice and Kras^Tg^;Sirt1^WT^ mice. These results were found because pneumocytes in Sirt1 Tg mice exhibited increased levels of tumor suppressor genes such as Gstm5, Sod3, Pygm, Timp2, Php3, Cav1, and Cdkn2c, whereas Sirt1 O/E decreased the levels of oncogenes such as Gfpt1, Aurkb, Prkci, and Madd during the early phases of Kras^G12V^ activation. In other words, Sirt1 O/E in pneumocytes can affect several tumor suppressor genes or oncogenes, which can indirectly affect Kras tumor development.

However, KRAS^Mut^-induced SIRT1 in our results directly regulates KRAS^Mut^ activity. We found that KRAS^Mut^-c-Myc axis-induced SIRT1 also bound to KRAS^Mut^ and deacetylated lysine 104 in KRAS^Mut^ in vitro and in vivo, which enhanced KRAS^Mut^ activity (Figs. 3 and 4). Therefore, SIRT1 K/D or SIRT1^H363Y^ expression increased KRAS^Mut^ acetylation, which decreased KRAS^Mut^ activity. We hypothesized that SIRT1 is a valuable target for KRAS^Mut^ lung cancer. In contrast to our hypothesis, SIRT1 K/D could not completely abolish the proliferation of KRAS^Mut^ lung cancer cells. SIRT1 K/D decreased cell proliferation and the colony number by less than 20% (Fig. 6A, B). Therefore, we thought that SIRT1 K/D may result in different secondary changes to KRAS^Mut^ downstream signaling and/or act through a feedback mechanism to compensate for the loss of oncogenic KRAS^Mut^ in lung cancer. The SIRT1 K/D-mediated decrease in KRAS^Mut^ activity decreased the phosphorylation levels of MEK and ERK. Although pMEK and pERK levels were decreased by SIRT1 K/D, the pAKT level was not reduced, which led us to investigate the upstream mediators of Akt. Interestingly, the phospho-EGFR level was increased through shedding of HB-EGF and AREG mediated by SIRT1 K/D-induced pAkt-pADAM17 activity in three types of KRAS^Mut^ cell lines. In addition, STAT3, which is an important downstream mediator of EGFR signaling and acts as a transcription factor to regulate oncogene expression, is activated in NSCLC cells^74,75^. These results demonstrate that the oncogenic KRAS^Mut^-induced downstream pathways are regulated by complex mechanisms. It is very difficult to explain how oncogenic KRAS^Mut^ controls all activities in the Ras/Raf-MAPK or PI3K-Akt pathways driving oncogenesis in KRAS^Mut^ lung cancer.

As mentioned above, many attempts have been made to block multiple aspects of KRAS^Mut^ activation: targeting GEFs and GAPs, targeting KRAS^Mut^ downstream proteins, KRAS^Mut^ K/D using nanomedicine and siRNA, and targeting PTMs of KRAS^Mut^. However, the development of KRAS^Mut^-targeted therapies is not easy or simple. In this study, maintaining the KRAS^Mut^ acetylation status decreased GEF-mediated nucleotide exchange and promoted the inactive GDP-bound state, resulting in decreased KRAS^Mut^ activity, which could offer possibilities for combination therapy with established chemotherapy agents for KRAS^Mut^ lung cancer. Therefore, we believe that the identification of regulatory enzymes of KRAS^Mut^ acetylation constitutes the first step toward the development of a new therapeutic strategy targeting PTMs of KRAS^Mut^. First, we found that the SIRT1 inhibitor EX527 showed a synergistic anticancer effect in combination with cisplatin or erlotinib. EX527 increased the acetylation of KRAS^Mut^ and thus diminished KRAS^Mut^ activity in H358 cells and the KRAS^Mut^ orthotropic lung cancer model (Figs. 6, 7, and 8). When EX527 was used in combination with cisplatin or erlotinib, the levels of apoptosis markers, cleaved PARP, cleaved caspase 3, PI-Annexin V staining, and Apo-BrdU TUNEL positivity were robustly increased compared with those in each single treatment group and the untreated group, as determined by western blotting and immunofluorescence staining. Based on these results, SIRT1 inhibitors could be candidate agents for combination therapy for KRAS^Mut^ lung cancer. Another method of maintaining KRAS^Mut^ acetylation is to identify the binding partner of the acetyltransferase for KRAS^Mut^. In this study, we found that the acetyltransferase for KRAS^Mut^ is p300 in the cytosol by an in vivo acetylation assay. Additionally, p300 was found to directly acetylate KRAS^Mut^ lysine 104 in an in vitro acetylation assay, and this result was confirmed by LC‒MS/MS (Fig. 5 and Supplementary Fig. 12). Although p300 is mainly a nuclear protein, it can be cytosolic, acting as an E4 ubiquitin ligase for p53^76^, and it is localized in the cytoplasm in breast carcinoma tissues but not in the adjacent normal mammary gland tissues^77^. The mechanism of KRAS^Mut^ acetylation by p300 in the cytosol remains unclear. Despite the p300 expression-dependent change in KRAS^Mut^ acetylation and activity, we did not investigate whether p300 activators such as CTPB or CCS1477 showed synergistic anti-KRAS^Mut^ cancer effects when combined with cisplatin or erlotinib.

In summary, *KRAS*^*Mut*^ NSCLC imposes an enormous health burden because of its high mortality. Although many clinical trials are being performed to explore potential therapeutic options, *KRAS*^*Mut*^ NSCLC remains refractory to all targeted monotherapies. Obviously, there is a tremendous need to develop new therapies to treat *KRAS*^*Mut*^ cancers. Our studies suggest that targeting PTM of the KRAS^Mut^ protein, specifically its acetylation, may constitute a new therapeutic strategy. KRAS^Mut^ lysine 104 is a very important posttranslationally modified residue that determines KRAS^Mut^ activity. Constitutively activated KRAS^Mut^ activates its downstream Raf-MEK-ERK-c-Myc axis, which increases the transcription and translation levels of SIRT1. KRAS^Mut^-induced SIRT1 stably deacetylates KRAS^Mut^ lysine 104. Consequently, the strong KRAS^Mut^-c-Myc-SIRT1 positive feedback loop may be a challenging therapeutic target. Because of this feedback loop, SIRT1 inhibition mediated by EX527 showed a synergistic anti-KRAS^Mut^ effect with cisplatin or erlotinib. In contrast, KRAS^Mut^ activity was noticeably reduced when p300 activity and KRAS^Mut^ lysine 104 acetylation were reinforced. These findings further support the potential of an effective combination regimen to sensitize KRAS^Mut^ NSCLC to clinical SIRT1 inhibitors or p300 activators via concurrent inhibition driven by either cisplatin or erlotinib.

### Supplementary information

Supplemental Figures and Tables
