# Supplementary material for: Oncogenic KRAS mutation confers chemoresistance by upregulating SIRT1 in non-small cell lung cancer
Source: Exp Mol Med. 2023 Oct 2;55(10):2220–37. doi: 10.1038/s12276-023-01091-0 (PMC10618295; doi:10.1038/s12276-023-01091-0)
Supplement: Supplementary file 1 — Supplemental Figures and Tables [file 12276_2023_1091_MOESM1_ESM.pdf]

Supplementary figure legends

Supplementary Figure. 1

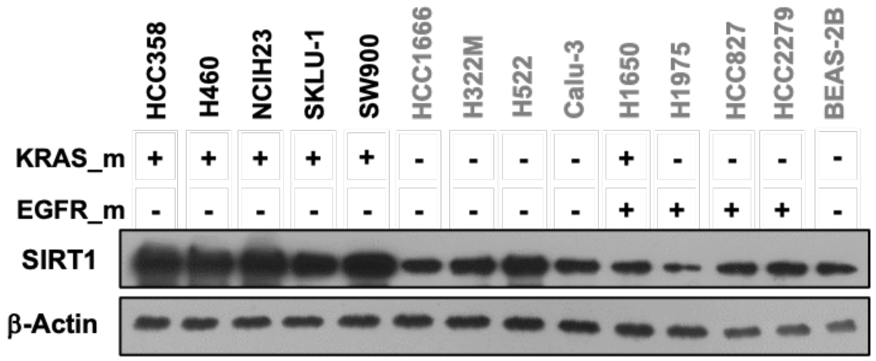

Supplementary Fig. 1 Measure SIRT1 protein expression in NSCLCs

SIRT1 protein expression in non-tumorigenic lung epithelial cells, $KRAS^{Mut}$ positive/negative cell lines, and $EGFR^{Mut}$ positive/negative cell lines.

Supplementary Figure. 2

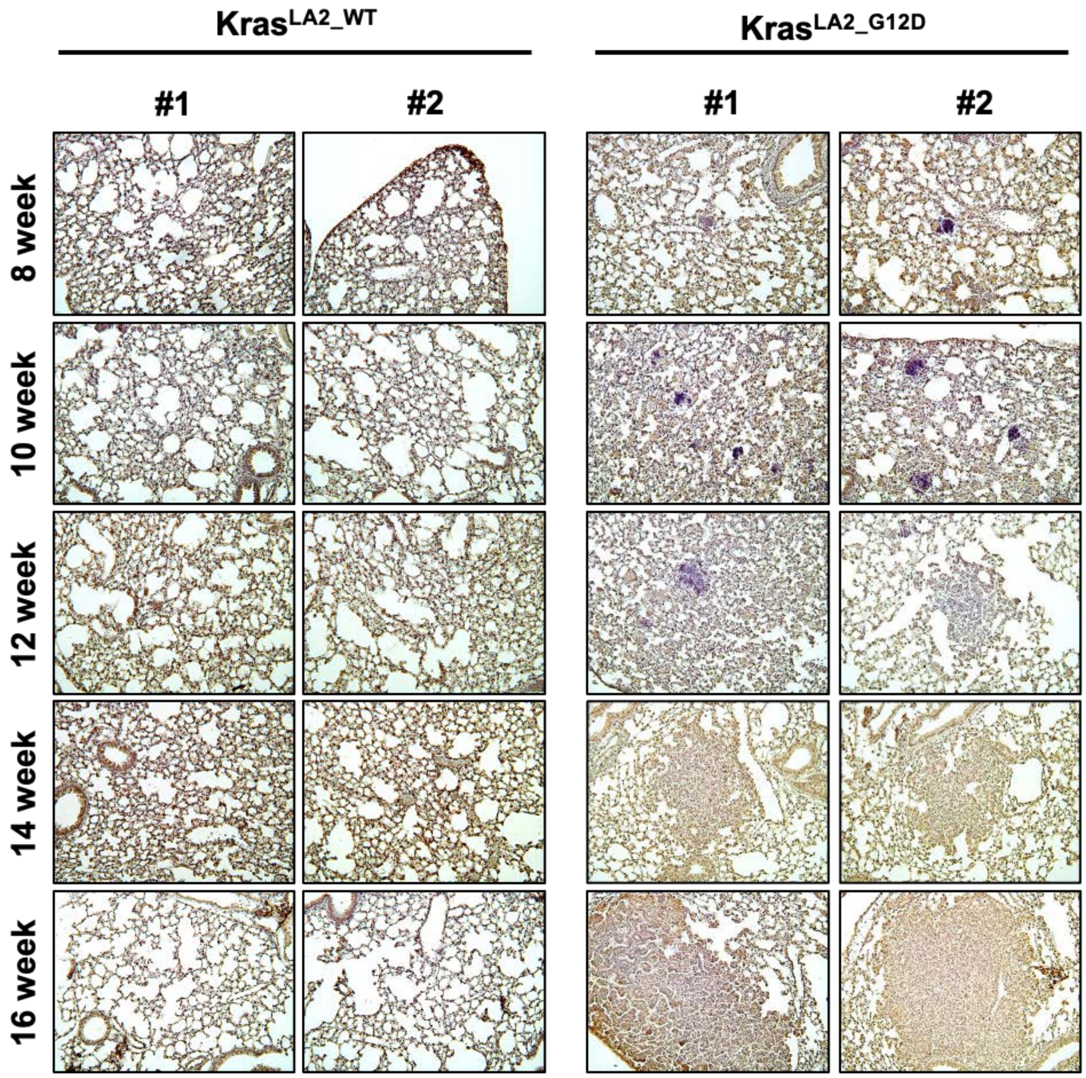

Supplementary Fig. 2 KRAS^{LA2_G12D}-driven lung cancer identification

H&E staining of lungs between 8 and 16 weeks after birth from KRAS^{LA2_WT} and KRAS^{LA2_G12D} mice.

Supplementary Figure. 3

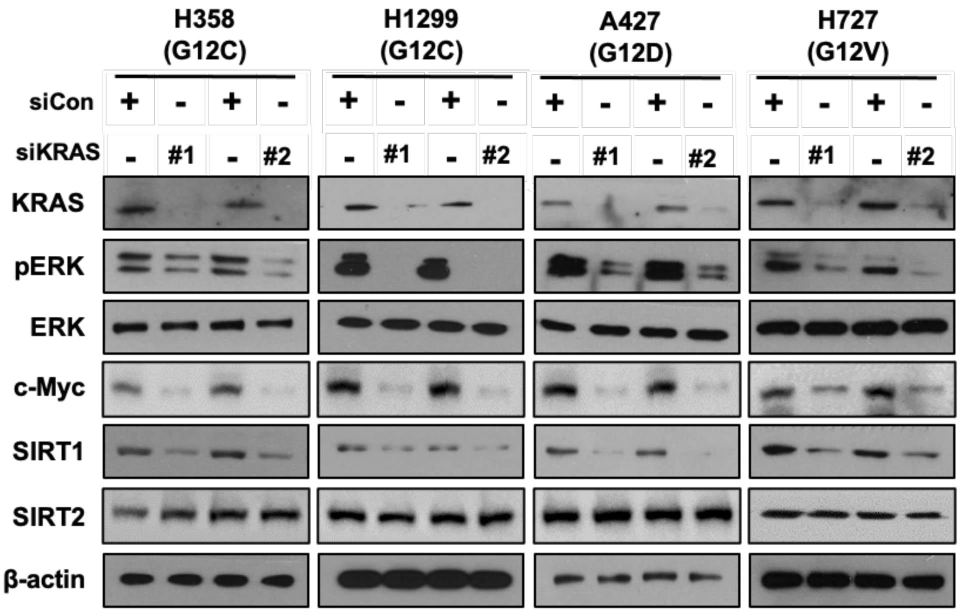

Supplementary Fig. 3 SIRT1 expression is associated with KRAS expression

HCC358, H1299 ($KRAS^{G12C}$ stable cell line), A427 ($KRAS^{G12D}$), and H727 ($KRAS^{G12V}$) cells were transfected with siCon, siKRAS (80 nM), pcDNA, $KRAS^{G12C}$, $KRAS^{G12D}$, and $KRAS^{G12V}$ (2 μg) plasmids. Cell lysates were immunoblotted with anti-KRAS, anti-SIRT1, anti-SIRT2, anti-c-Myc, anti-pERK, anti-ERK, and anti-β-actin antibodies after 72 h after transfection.

Supplementary Figure. 4

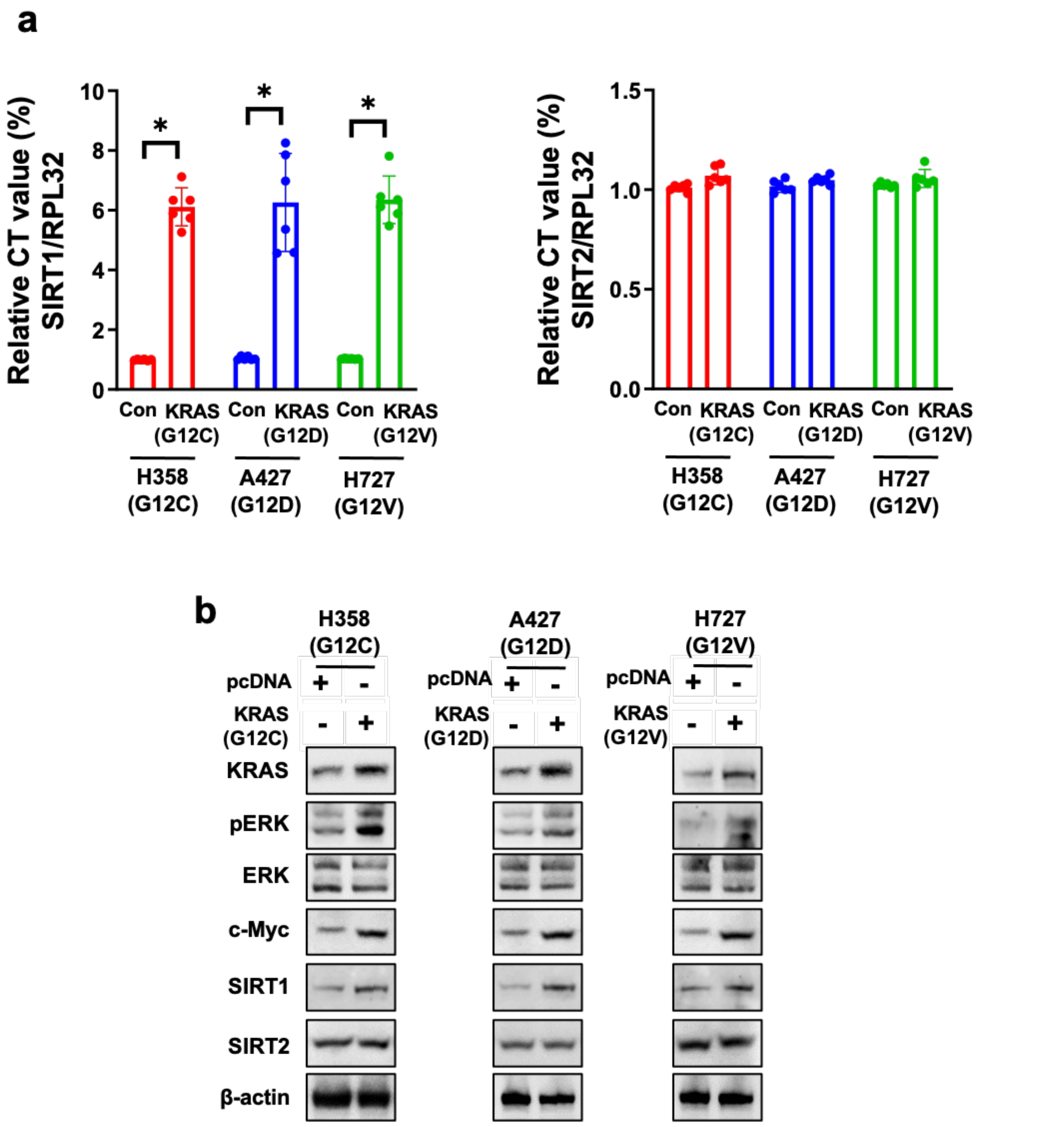

Supplementary Fig. 4 Identification of SIRT1 and SIRT2 expression in KRAS^{Mut} cells

a The mRNA expression of SIRT1 and SIRT2 was measured by quantitative real-time PCR with H358, A427, and H727 cell lines of Supplementary Fig. 3. *RPL32* was used as internal control and for normalization. Student's *t*-test, mean ± SD; n = 6, *, $p < 0.05$. **b** The previous three cell lines were transfected with pcDNA, KRAS^{G12C}, KRAS^{G12D}, and KRAS^{G12V} (2 μg) plasmids. Cell lysates were immunoblotted with anti-KRAS, anti-SIRT1, anti-SIRT2, anti-c-Myc, anti-pERK, anti-ERK, and anti-β-actin antibodies after 72 h after transfection.

Supplementary Figure. 5

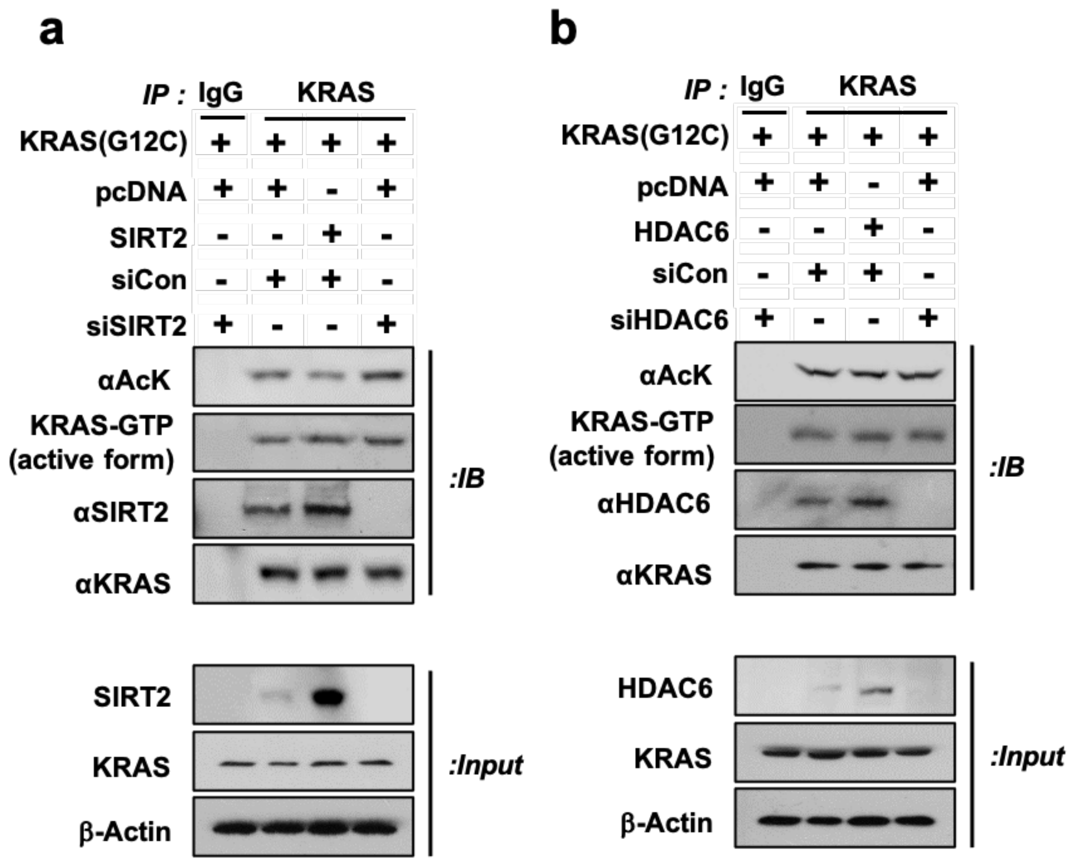

Supplementary Fig. 5 Identification of the KRAS^{Mut} activity regulated by SIRT2 and HDAC6

a, **b** Plasmid (*pcDNA*, *KRAS^{G12C}*, *SIRT2,* and *HDAC6* each 4 μg) and siRNA (siCon and siSIRT1 each 80 nM) were transfected into H358 cells. Cell extracts were immunoprecipitated by anti-KRAS antibody and Raf-1 agarose beads, and analyzed using anti-acetylation, anti-SIRT2, anti-HDAC6, anti-KRAS, and anti-KRAS-GTP bound antibody.

Supplementary Figure. 6

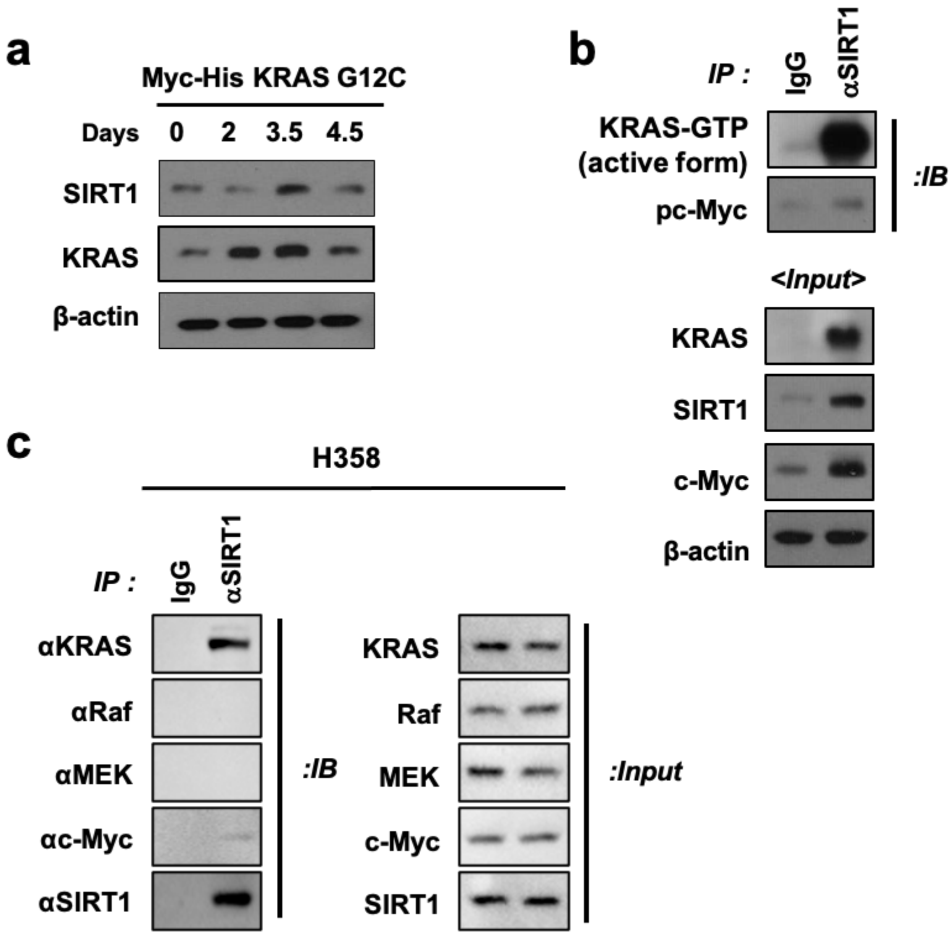

Supplementary Fig. 6 KRAS^{Mut}-induced SIRT1 more binds to KRAS^{Mut} than to c-Myc

a HEK293T cells were expressing Myc-His-KRAS^{G12C}, and then the cells were harvested with lysis buffer on the indicated day and subjected to western blotting. **b** Cell lysates at 3.5 day under the same conditions as **(a)** were immunoprecipitated with anti-SIRT1 antibody and anti-KRAS-GTP and p-c-Myc antibodies were immunoblotted. **c** H358 cell lysates were immunoprecipitated with anti-SIRT1 and anti-KRAS, anti-Raf, anti-MEK, anti-c-Myc, and anti-SIRT1 antibodies were used to do immunoblot analysis.

Supplementary Figure. 7

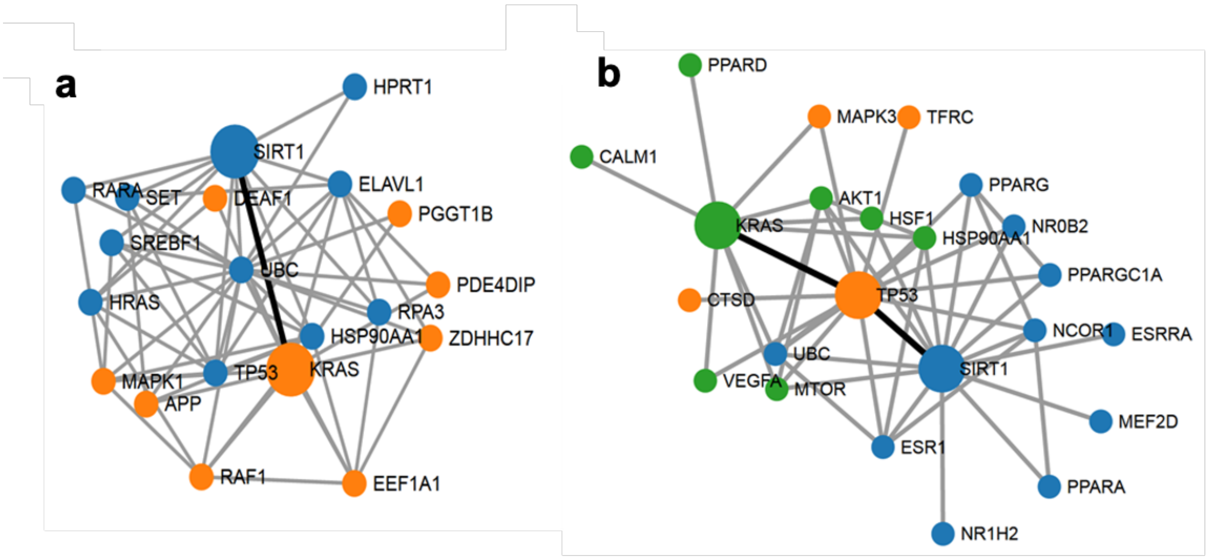

Supplementary Fig. 7 Shortest path between KRAS^{WT} and SIRT1 was analyzed using PINA and STRING in ASEB

a, b Interactive relationships of two proteins KRAS^{WT} and SIRT1, were analyzed using PINA **(a)** and STRING **(b)** analysis provided by ASEB.

Supplementary Figure. 8

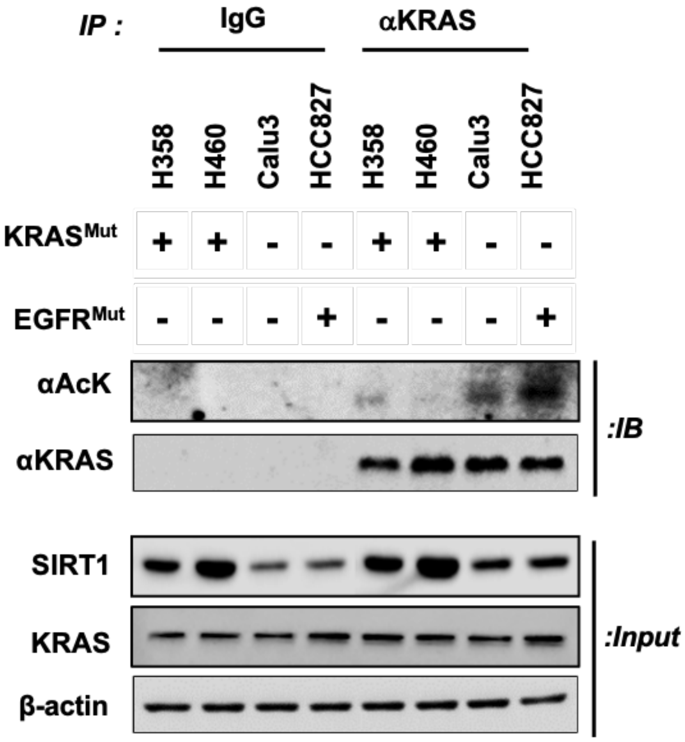

Supplementary Fig.8 The comparison of acetylation status between KRAS^{Mut}, EGFR^{Mut} , and both non-mutation cells.

Each KRAS^{Mut}, EGFR^{Mut}, and both negative NSCLCs lysates were immunoprecipitated with anti-KRAS and analyzed by antibody against acetyl-lysine and anti-KRAS antibody.

Supplementary Figure. 9

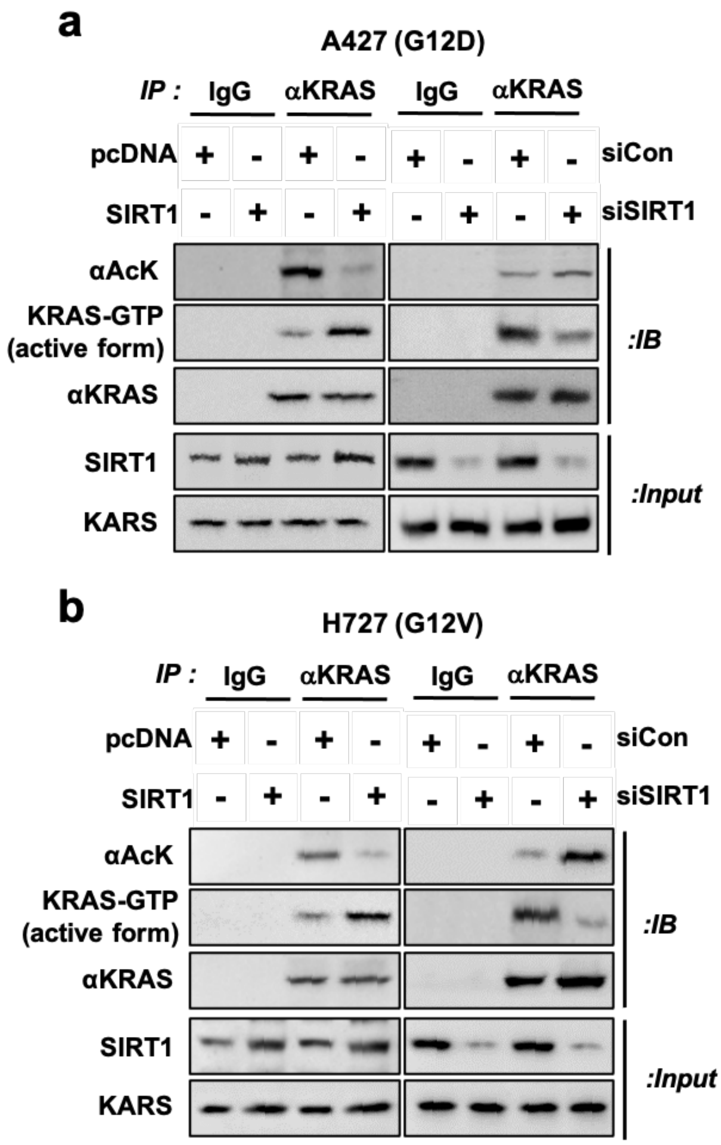

Supplementary Fig. 9 SIRT1 deacetylated and increased the activity of KRAS^{G12D} and KRAS^{G12V} as well as KRAS^{G12C}.

a, b A427 (KRAS^{G12D}) and H727 (KRAS^{G12V}) cells were transfected with siCon, siSIRT1 (80 nM), pcDNA, SIRT1 plasmids (4 μg). Cell lysates were subjected to immunoprecipitation with anti-KRAS and then analyzed using anti-acetyl lysin, anti-KRAS-GTP, anti-KRAS, and anti-SIRT1 antibodies after 72 h after transfection.

Supplementary Figure. 10

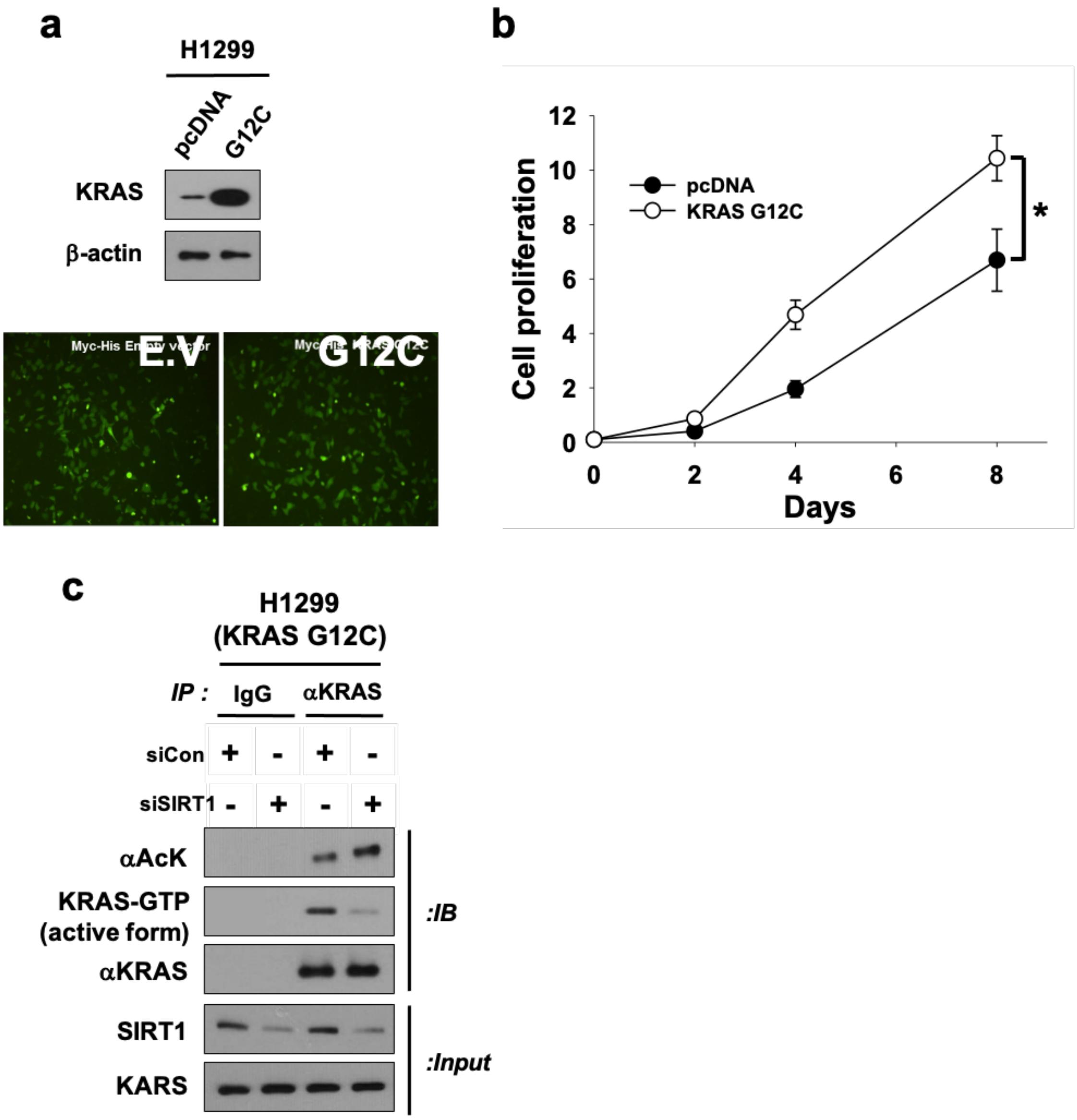

Supplementary Fig.10 Established H1299 KRAS^{G12C} stable cell line validated SIRT1 regulation of KRAS^{G12C} acetylation and cell proliferation

a H1299 (KRAS^{WT}) cells were transformed by stably expressing KRAS^{G12C}. Selected stable cells treated with G418 (400 μg/ml) for two weeks were identified by GFP fluorescence. **b** The growth rates of H1299 KRAS^{G12C} stable cells were measured using MTS assay. Student's *t*-test, mean ± SD; n = 6, *, $p < 0.05$. **c** H1299 KRAS^{G12C} stable cell lines were transfected with siCon and siSIRT1 (80 nM) for 72 h. Anti-IgG and anti-KRAS antibodies were immunoprecipitated and immunoblotted with anti-acetyl lysine, anti-KRAS-GTP, anti-KRAS, and anti-SIRT1 antibodies.

Supplementary Figure. 11

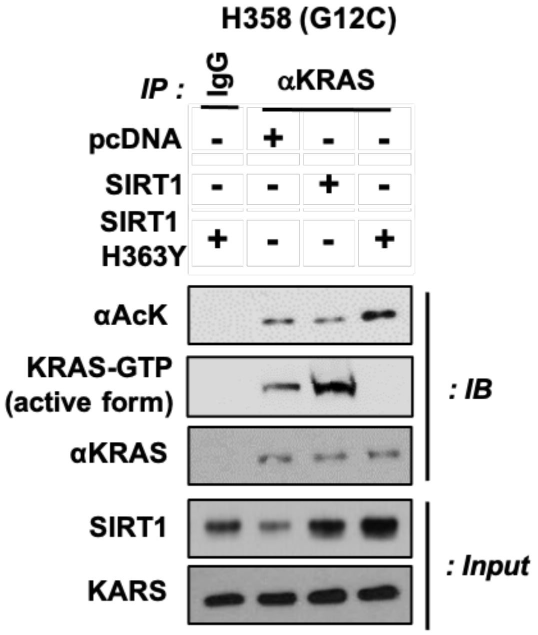

Supplementary Fig.11 SIRT1 deacetylases KRAS^{Mut}

H358 cells were transfected with the following plasmids: pcDNA, SIRT1, and SIRT1^{H363Y} (a catalytically impaired inactive SIRT1 mutant) (4 μg). Cell lysates were subjected to immunoprecipitation with anti-KRAS and, then analyzed using anti-acetyl lysin and anti-KRAS antibodies.

Supplementary Figure. 12

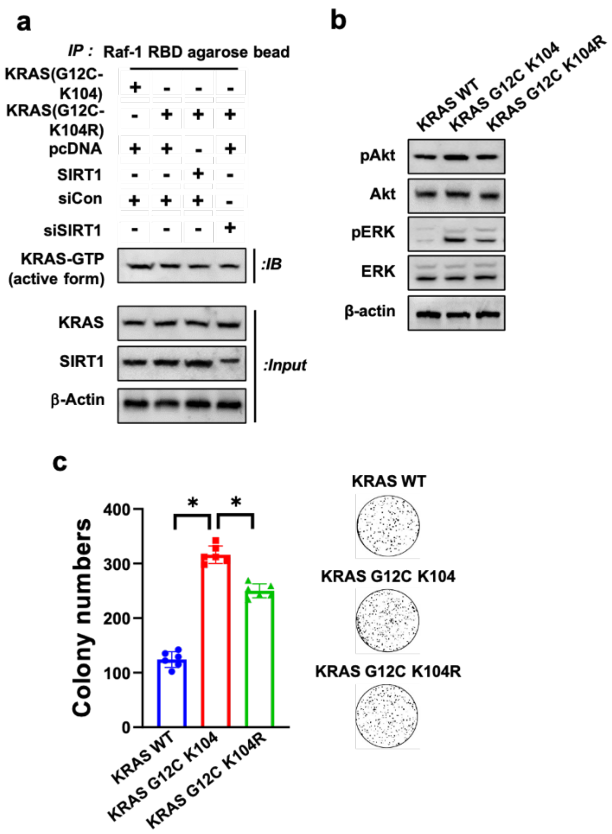

Supplementary Fig.12 The comparison of oncogenic activity between KRAS^{G12C_K014} and KRAS^{G12C_K104R}

a H1299 (KRAS^{WT}) cells were transformed by stably expression KRAS^{G12C_K104R}. Selected stable cells treated with G418 (400 ug/ml) for two weeks were identified by GFP expression. H1299 KRAS^{G12C_K104} and H1299 KRAS^{G12C_K104R} stable cell lines were transfected with *pcDNA* and *SIRT1* each 4 μg and siCon and siSIRT1 each 80 nM. Each cell extracts were immunoprecipitated by Raf-1 agarose beads, and analyzed using anti-KRAS-GTP bound antibody. **b** The cell lysates from H1299 KRAS^{WT}, H1299 KRAS^{G12C_K104}, and H1299 KRAS^{G12C_K104R} stable cell lines were analyzed by western blotting with anti-pAKT, anti-Akt, anti-pERK, anti-ERK, and anti-β-actin antibodies. **c** Three H1299 stable cell lines were seeded with 0.5% top agar and cultured in a mixture of fresh medium. Cell colonies were stained with crystal violet and counted per 3.8 cm^2 (Left panel). Representative colony images were showed (Right panel). Student's *t*-test, mean ± SD; n = 6, *, $p < 0.05$.

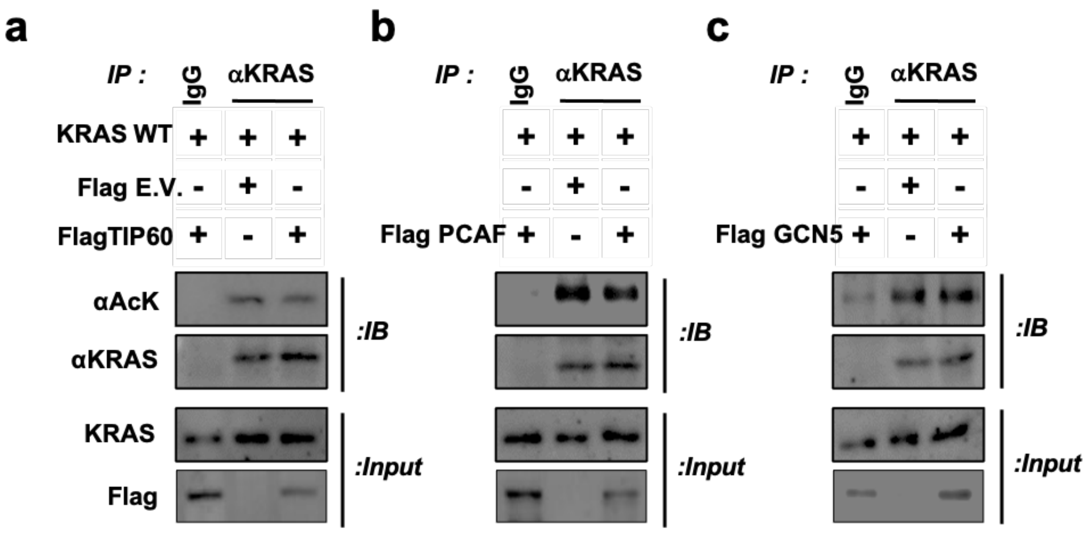

Supplementary Fig.13 Identification of KRAS acetylase enzyme

a-c HEK293T cells were transfected with $KRAS^{G12C}$, Flag-E.V., and Flag-TIP60/PCAF/GCN5 plasmids. Cell lysates were immunoprecipitated with anti-IgG and anti-KRAS antibodies and then immunoblotted with anti-acetyl lysine, anti-Flag, and anti-KRAS antibodies.

Supplementary Figure. 14

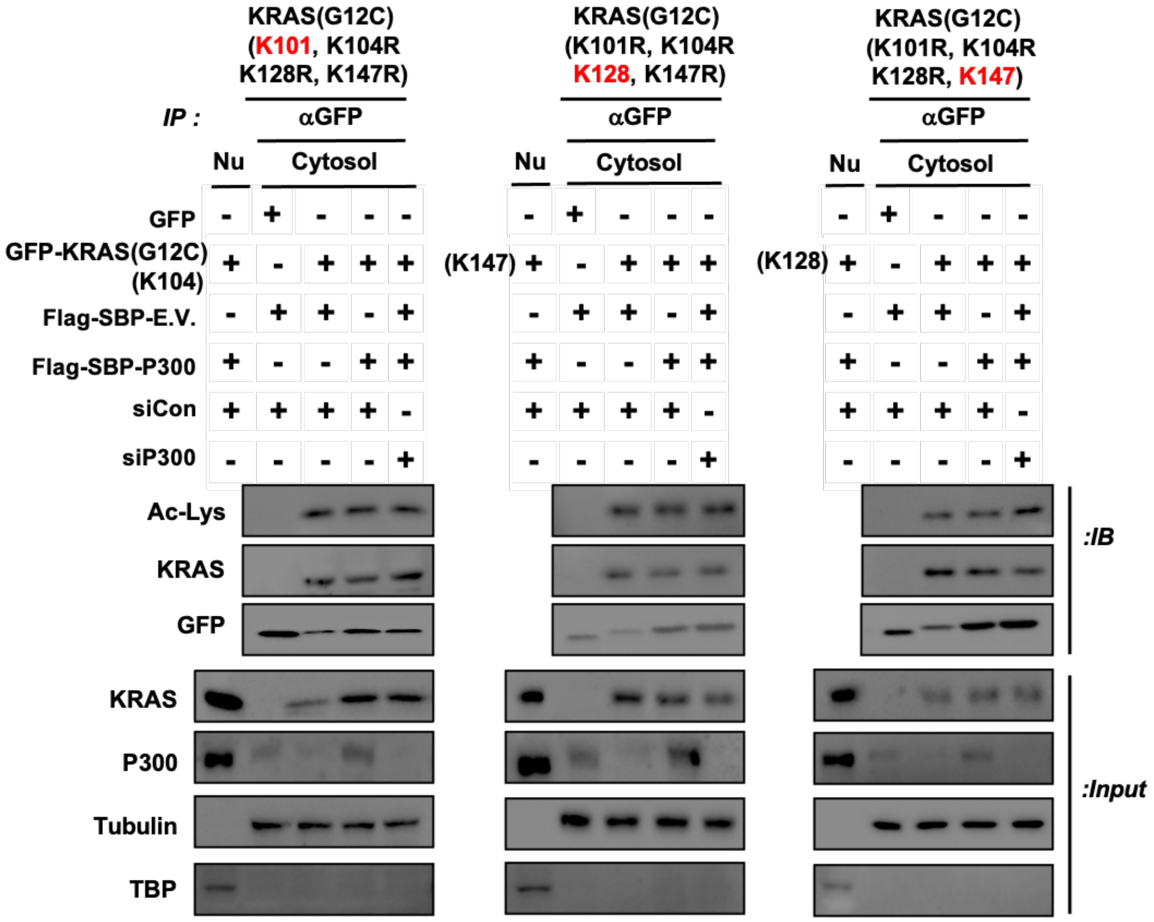

Supplementary Fig.14 KRAS-K101, K128, and K147 are not acetylation sites for the p300 acetylase enzyme

HEK293T cells were transfected with GFP-E.V., GFP-KRAS (acetylated lysin residues [K101, K104, K128, and K147] are changed by three arginines in regular sequence), Flag-SBP-E.V., Flag-SBP-p300, siCon, and sip300. Cell lysates were immunoprecipitated with an anti-GFP antibody and then analyzed using anti-acetyl lysine, anti-KRAS, and anti-GFP antibodies.

Supplementary Figure. 15

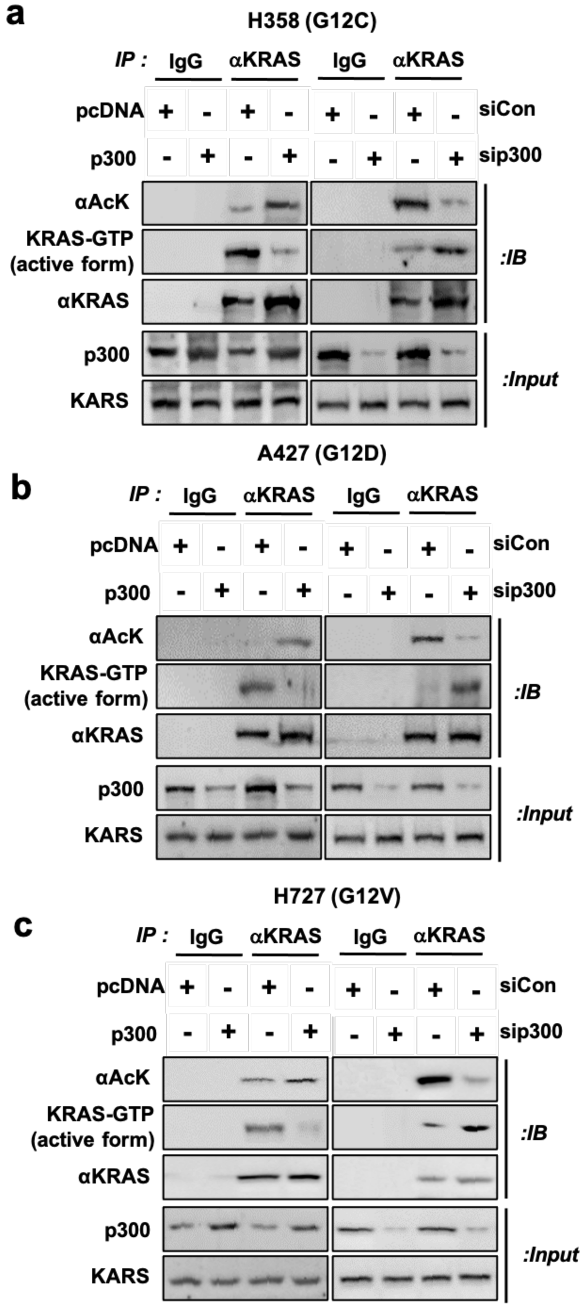

Supplementary Fig.15 Acetyltransferase p300 acetylated and decreased the activity of KRAS^{G12C}, KRAS^{G12D}, and KRAS^{G12V}.

a-c A358 (KRAS^{G12D}), A427 (KRAS^{G12D}), and H727 (KRAS^{G12V}) cells were transfected with siCon, sip300 (80 nM), pcDNA, p300 plasmids (4 μg). Cell lysates were subjected to immunoprecipitation with anti-KRAS and then analyzed by immunoblotting using anti-acetyl lysin, anti-KRAS-GTP, anti-KRAS, and anti-p300 antibodies after 72 h after transfection.

Supplementary Figure. 16

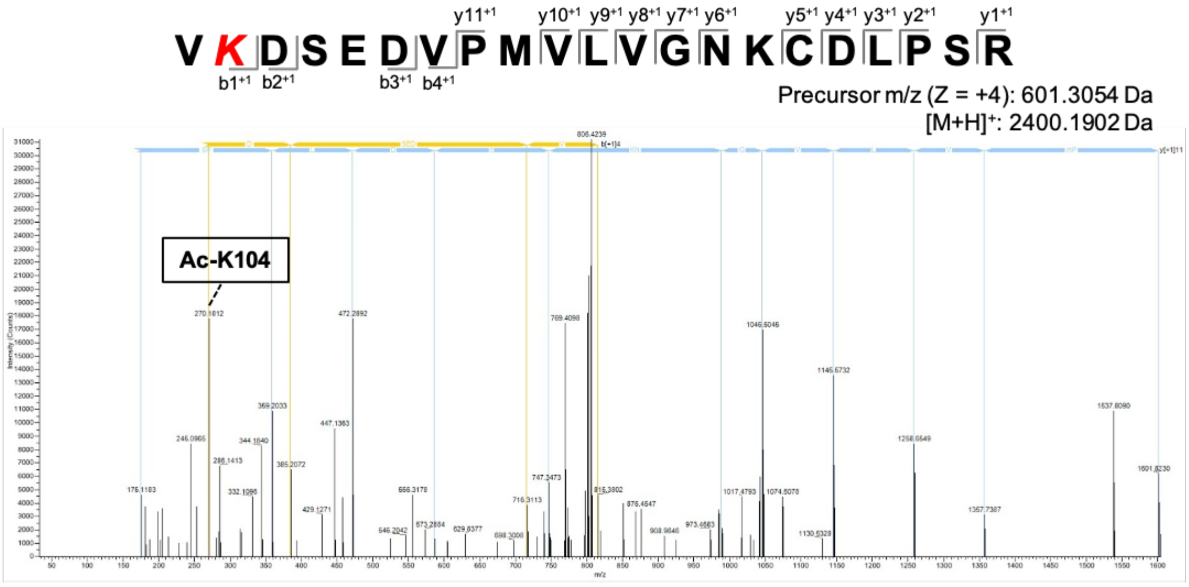

Supplementary Fig.16 LC-MS/MS data from the *in vitro* acetylation and deacetylation assay

The MS/MS spectrum of the KRAS^{G12C} peptide a.a. 103-VKDSEDVPMVLVGNKCDLPSR-123 is shown **Fig. 5e** (3) sample. The mass spectrum composed of fragmentation of the ion at m/z 2400.1902 refers to the acetylation of the KRAS^{G12C} peptide a.a. 103-123. A novel lysine acetylation site for residue K104 in the KRAS^{G12C} peptide is indicated. A series of fragments extending from the N-terminus are b ions, and a series of fragments extending from the C terminus are y ions.

Supplementary Figure. 17

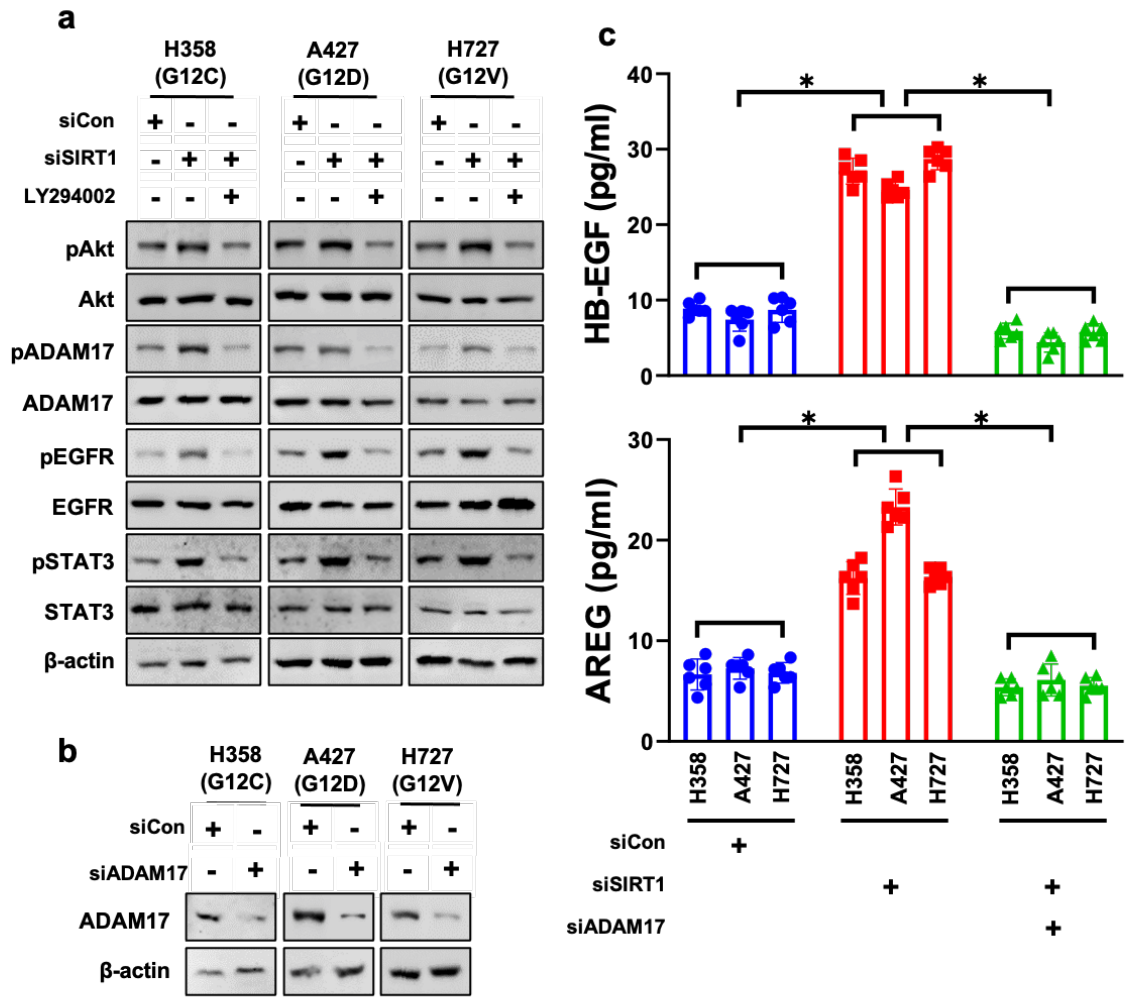

Supplementary Fig.17 SIRT1 K/D-induced EGFR activation is due to increased heparin-binding EGF (HB-EGF) and amphiregulin shed by ADAM17.

a H358 ($KRAS^{G12D}$), A427 ($KRAS^{G12D}$), and H727 ($KRAS^{G12V}$) cells were transfected with siCon and siSIRT1 (80 nM) treated with LY294002 (50 μM). The lysates from three cell lines were immunoblotted by anti-pAkt, anti-Akt, anti-pADAM17, anti-ADMA17, anti-pEGFR, anti-EGFR, anti-pSTAT3, and anti-STAT-3, and anti-β-actin antibodies. **b** siCon and siADAM17 (80 nM) were transfected into H358, A427, and H727 cells and then immunoblotted using anti-ADAM17 and anti-β-actin antibodies. **c** H358, A427, and H727 cells were transfected with siCon, siSIRT1, and siADAM17. The supernatant medium was collected and HB-EGF and AREG ELISA was performed to measure the levels of HB-EGF and AREG. Student's *t*-test, mean ± SD; n = 6, *, $p < 0.05$.

Supplementary Figure. 18

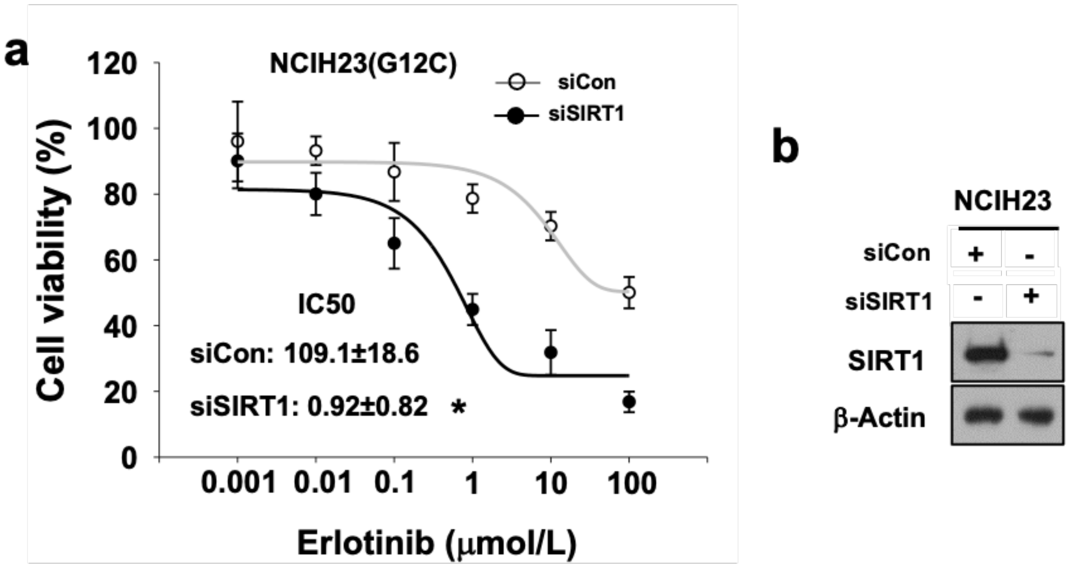

Supplementary Fig.18 Check the synergistic anti-proliferative effect between SIRT1 inhibition and erlotinib in NCIH23 cells

a NICH23 cells were transfected with siCon and siSIRT1 (80 nM) and then seeded 96 well contained with erlotinib at concentrations between 0.001 and 100 μM. Cell viability was measured using MTS assay. Student's *t*-test, mean ± SD; n = 6, *, $p < 0.05$. **b** SIRT1 K/D expression in transfected cells was measured by western blotting.

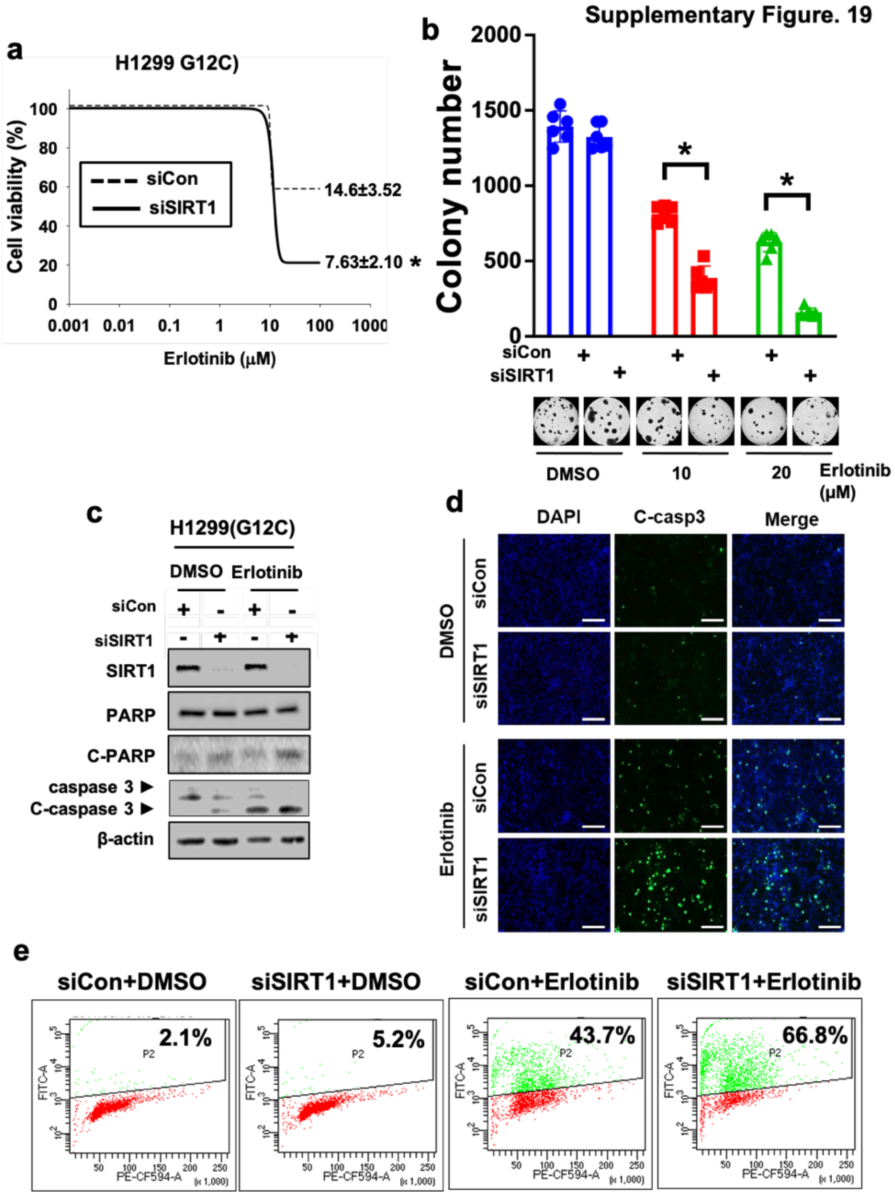

Supplementary Fig.19 SIRT1 K/D supported the synergistic anti-cancer effects of erlotinib on the KRAS^{G12C} stable cell line

a, **b** H1299 KRAS^{G12C} stable cells, as shown in **Supplementary Fig. S10** were transfected with siCon and siSIRT1 (80 nM) and then seeded 96 well **(a)** for growth curve and 12 wells into agarose **(b)** for colony formation assay with dose dependent treatment with erlotinib. Student's *t*-test, mean ± SD; n = 6, *, $p < 0.05$. **c-e,** H1299 KRAS^{G12C} stable cells were transfected with siCon and siSIRT1 (80 nM), and then treated with erlotinib (10 μM). **c** Western blotting was performed by anti-PARP and caspase-3 antibody. **d** Cells treated with erlotinib were subjected to immunofluorescence staining, and **e** DNA damage analysis using the APO-BrdU TUNEL assay.

Supplementary Figure. 20

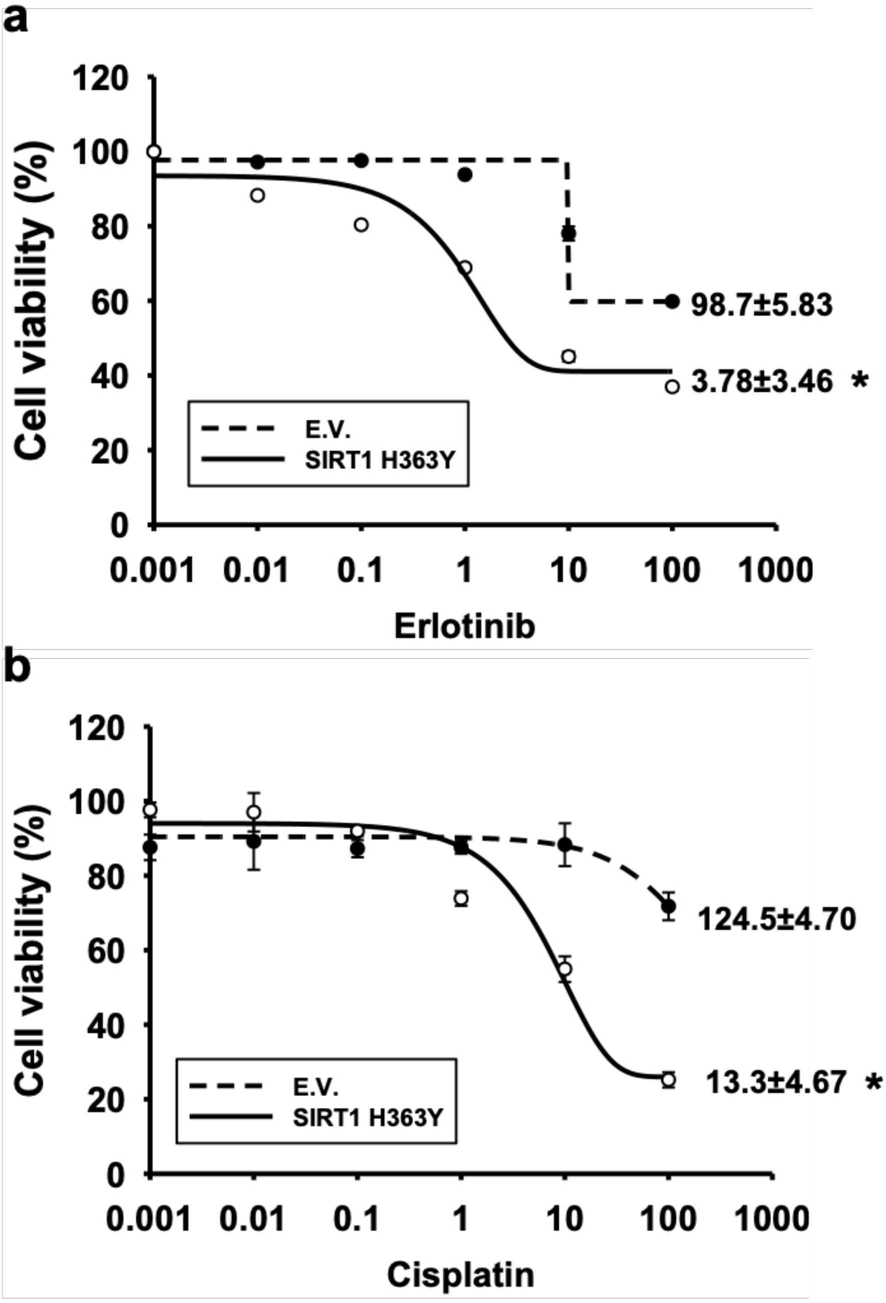

Supplementary Fig. 20 Check the synergistic anti-cancer viability effect of cisplatin and erlotinib on the H358^{SIRT1_H363Y} stable cell line.

a, b H358^{SIRT1_H363Y} stable cells were treated with DMSO and cisplatin **(a)** and erlotinib **(b)** in a dose-dependent manner, and cell viability was measured using the MTS assay.

Supplementary Figure. 21

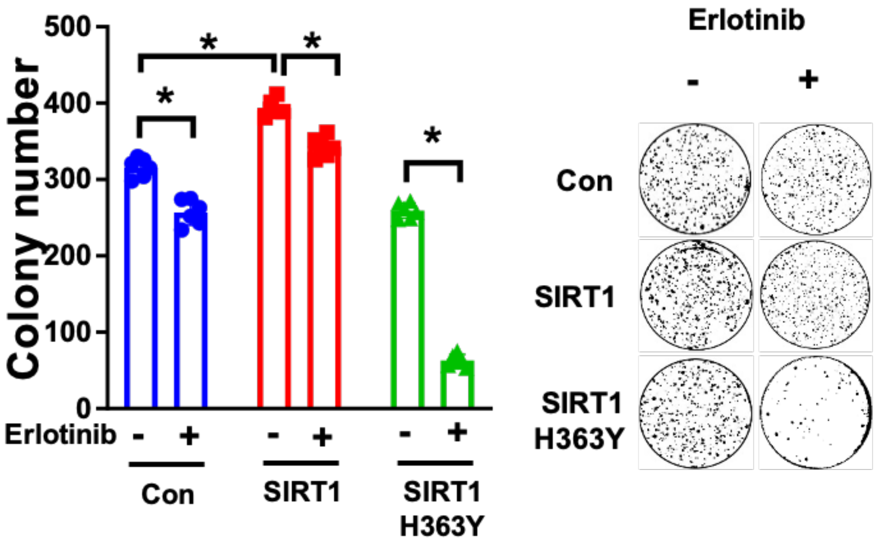

Supplementary Fig. 21 The comparison of tumorigenicity ability between control, SIRT1 O/E, and SIRT1 H363Y

H358^{SIRT1_WT}, H358$^{SIRT1_O/E}$, and H358^{SIRT1_H363Y} stable cells were seeded with 0.5% top agar and cultured in a mixture of medium and erlotinib (10 μM). Cell colonies were stained with crystal violet and counted per 3.8 cm^2. Student's *t*-test, mean ± SD; n = 6, *, $p < 0.05$.

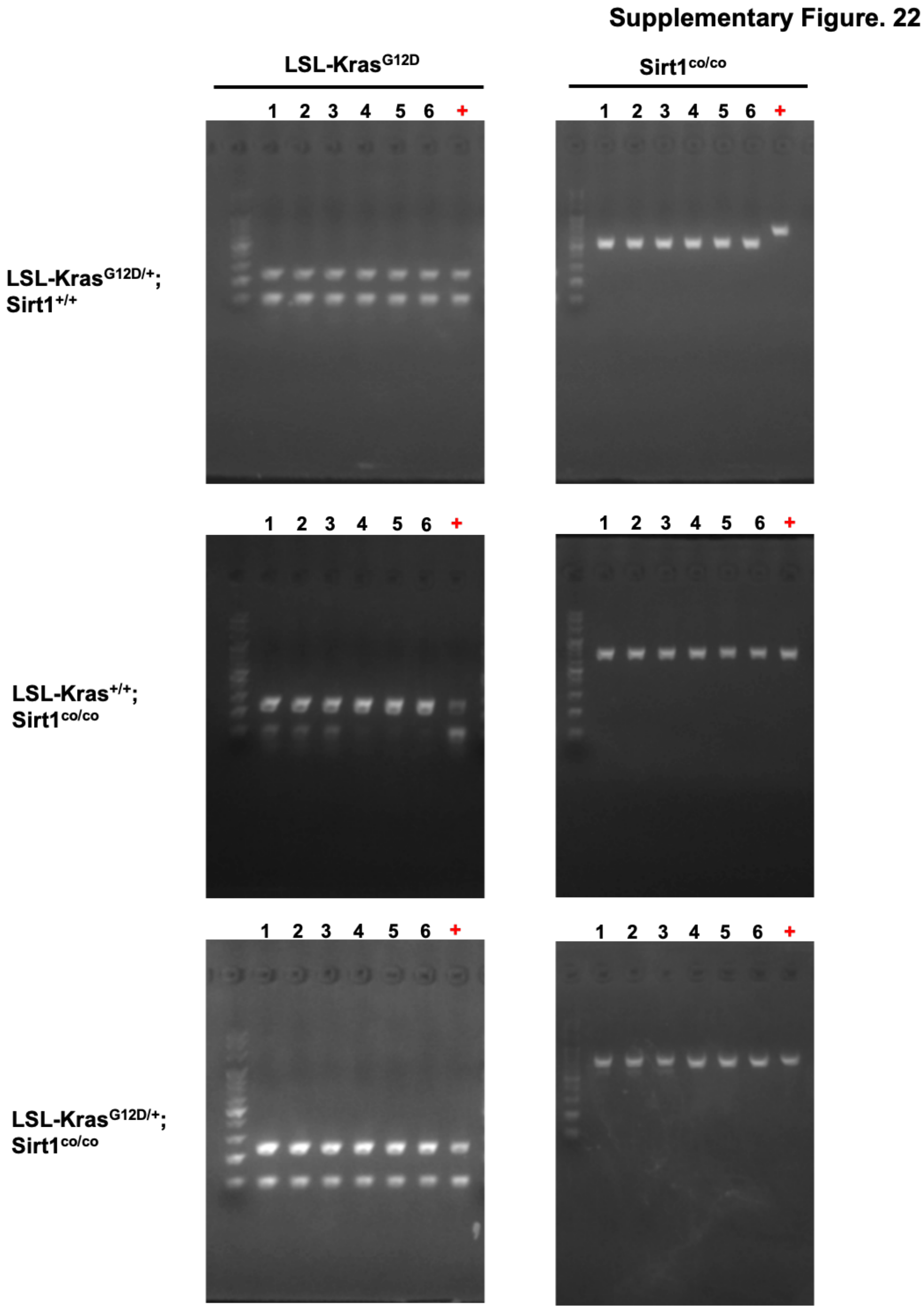

Supplementary Fig. 22 Genotyping PCR analysis

The identification of LSL-Kras$^{G12D/+}$;Sirt1$^{+/+}$ and LSL-Kras$^{G12D/+}$;Sirt1$^{co/co}$ mice via Genotyping PCR analysis

Supplementary Figure. 23

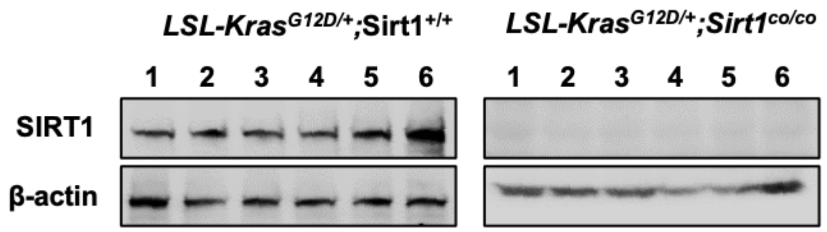

Supplementary Fig. 23 SIRT1 K/D identification from LSL-Kras$^{G12D/+}$;Sirt1$^{+/+}$ and LSL-Kras$^{G12D/+}$; Sirt1$^{co/co}$ mouse lungs

Immunoblotting analysis of SIRT1 and β-actin (loading control) expression in lung lysates from LSL-Kras$^{G12D/+}$;Sirt1$^{+/+}$ and LSL-Kras$^{G12D/+}$;Sirt1$^{co/co}$ mice.

Supplementary Figure. 24

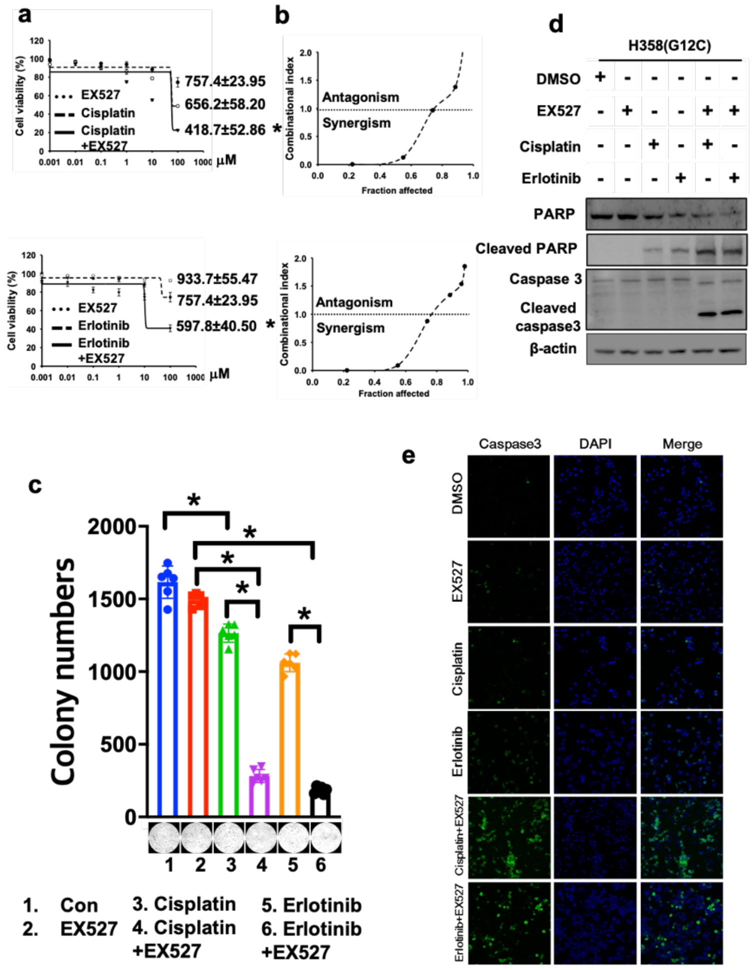

Supplementary Fig. 24 SIRT1 inhibitor is required for the synergistic therapeutic efficacy of EGFR TKI in $KRAS^{G12C}$ lung tumor cells

a, b SIRT1 inhibitor augments the inhibitory effects of cisplatin and erlotinib on $KRAS^{Mut}$ cell (H358 cells), as indicated by **(a)** growth curves and **(b)** analysis of combined drug effects. Drug synergy was determined by the combination index (CI) analysis according to the median-effect method of Chou and Talalay using the CalcuSyn software. The CI is a quantitative representation of the degree of drug Interaction. **c-e,** H358 cells were treated with EX527 (10 μM) in combination with cisplatin (10 μM) or erlotinib (10 μM), as indicated by **(c)** measured colony-forming capabilities. Data shown are representative images of soft agar assays and relative colony numbers in cells treated with the indicated drugs after 14 days of incubation. Student's *t*-test, mean ± SD; n = 6, *, $p < 0.05$. cell contained with drugs were measured apoptosis markers by using western blotting **(d)** and immunofluorescence staining **(e)** after 72 h of drugs' treatment.

Supplementary Figure. 25

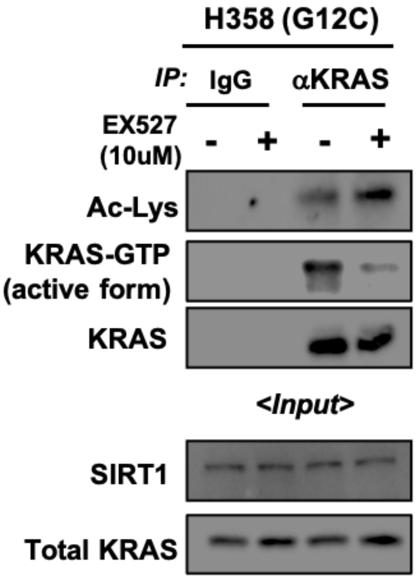

Supplementary Fig. 25 SIRT1 inhibitor, EX527 increased an acetylation of $KRAS^{Mut}$

H358 cells were treated with EX527 10 μM and then the cell lysates were immunoprecipitated with anti-IgG and anti-KRAS antibodies and then immunoblotted with anti-acetyl lysine, anti-KRAS-GTP, and anti-KRAS antibodies.

Supplementary Figure. 26

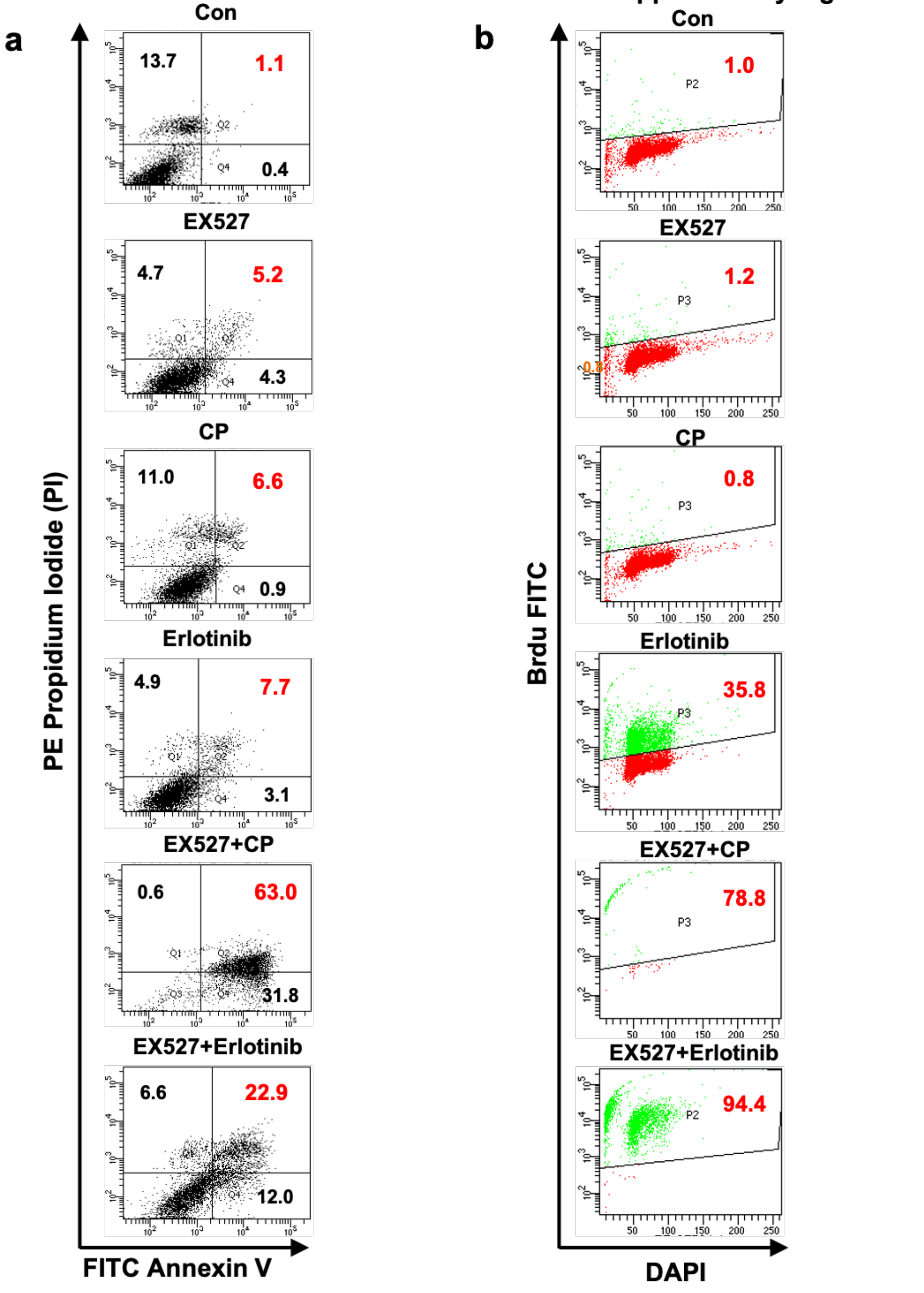

Supplementary Fig. 26 The SIRT1 inhibitor, EX527 augmented apoptosis of KRAS mutant cells treated with cisplatin or erlotinib

a, b H358 cells were treated with DMSO, EX527 (10 μM), cisplatin (10 μM), erlotinib, and combined EX527 with cisplatin or erlotinib, and then FACS analysis was performed for PI & Annexin V staining (a) and Apo-BrdU TUNEL staining (b).

Supplementary Figure. 27

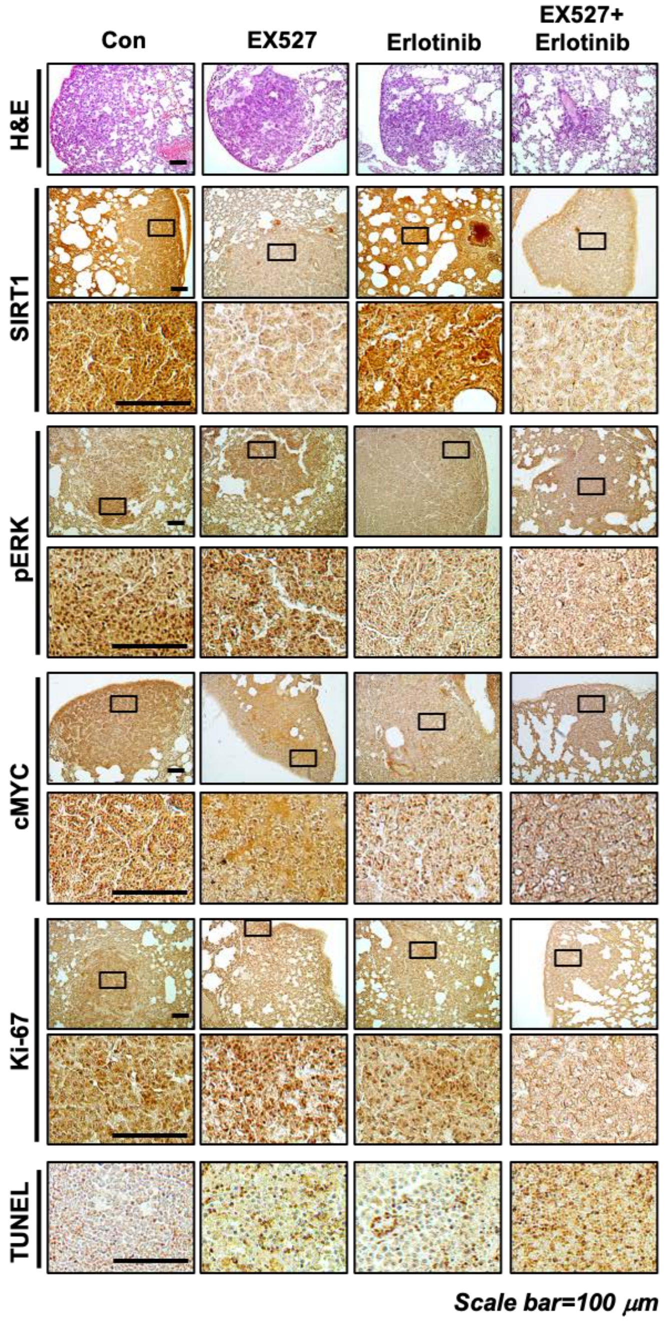

Supplementary Fig. 27 Identification of the synergistic anti-cancer effect of erlotinib in the KRAS^{Mut} lung orthotopic model

Immunohistochemical staining of mouse lung tumor tissues was performed using anti-SRIT1, anti-pERK, anti-c-Myc, and anti-Ki-67 antibodies. Representative images are shown. Scale bar, 100 μm. High-magnification images correspond to areas marked by the black box.

Supplementary table legends

Supplementary Table. 1

Table 1. The activity of proteins deacetylated by SIRT1.

Protein	Site	Sequence	Activity	Reference
TRP53	Lys-382	HLKSKKGQSTS	Inactivation	Hori et al.
RELA/NF-kBp65	Lys-310	TYETFKSIMKK	Inactivation	Yeung et al.
HIF-1α	Lys-674	IEQTEKSHPRS	Inactivation	Lim et al.
PML	Lys-487	TTTAQKRKCSQ	Inactivation	Guan et al.
XRCC6/Ku70	Lys-539	YNPEGKVTKRK	Activation	Jeong et al
LKB1	Lys-48	AKLIGKYLMGD	Activation	Lan et al.
KRAS	Lys-104	QIKRVKDSEDV	Activation	

Supplementary Table. 1 Activity of proteins deacetylated by SIRT1

The proteins, TRP53, NF-κBp65, HIF-1α, PML, XRCC6/ku70, and LKB1 are deacetylated by SIRT1, and changes in their activity are described.

Table 2. Prediction of deacetylation of KRAS^{WT} with ASEB P-values for candidate sites

Enzyme	Site	Sequence	P-value *	
**SIRT1**	**104**	YREQKRV**K** DSEDVPMV	**0.5089 (Top 39%)**	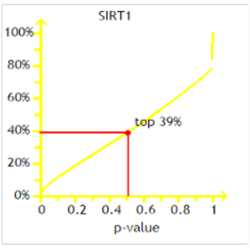
HDAC1/ HDAC2/ HDAC3	104	YREQIKRV**K** DSEDVPMV	**0.491 (Top 41%)**	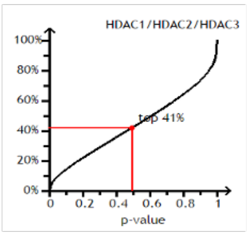

*** P-values for query peptides were represented**

Supplementary Table. 2 Prediction of deacetylation of KRAS^{WT} with ASEB P-values for candidate sites

Deacetylation sites of KRAS^{WT} were explored K104 lysine by SIRT1 (*p*-value 0.5089, top 39%) and by HDAC1/HDAC2/HDAC3 (*p*-value 0.4941, top 41%).
